# metaProbiotics: a tool for mining probiotic from metagenomic binning data based on a language model

**DOI:** 10.1093/bib/bbae085

**Published:** 2024-03-14

**Authors:** Shufang Wu, Tao Feng, Waijiao Tang, Cancan Qi, Jie Gao, Xiaolong He, Jiaxuan Wang, Hongwei Zhou, Zhencheng Fang

**Affiliations:** Microbiome Medicine Center, Department of Laboratory Medicine, Zhujiang Hospital, Southern Medical University, Guangzhou, China; Microbiome Medicine Center, Department of Laboratory Medicine, Zhujiang Hospital, Southern Medical University, Guangzhou, China; Microbiome Medicine Center, Department of Laboratory Medicine, Zhujiang Hospital, Southern Medical University, Guangzhou, China; Microbiome Medicine Center, Department of Laboratory Medicine, Zhujiang Hospital, Southern Medical University, Guangzhou, China; Microbiome Medicine Center, Department of Laboratory Medicine, Zhujiang Hospital, Southern Medical University, Guangzhou, China; Department of Gastroenterology, The Second Affiliated Hospital of Guangzhou Medical University, Guangzhou, China; Microbiome Medicine Center, Department of Laboratory Medicine, Zhujiang Hospital, Southern Medical University, Guangzhou, China; Microbiome Medicine Center, Department of Laboratory Medicine, Zhujiang Hospital, Southern Medical University, Guangzhou, China; Microbiome Medicine Center, Department of Laboratory Medicine, Zhujiang Hospital, Southern Medical University, Guangzhou, China; Microbiome Medicine Center, Department of Laboratory Medicine, Zhujiang Hospital, Southern Medical University, Guangzhou, China

**Keywords:** probiotics, metagenome, language model, machine learning

## Abstract

Beneficial bacteria remain largely unexplored. Lacking systematic methods, understanding probiotic community traits becomes challenging, leading to various conclusions about their probiotic effects among different publications. We developed language model–based metaProbiotics to rapidly detect probiotic bins from metagenomes, demonstrating superior performance in simulated benchmark datasets. Testing on gut metagenomes from probiotic-treated individuals, it revealed the probioticity of intervention strains–derived bins and other probiotic-associated bins beyond the training data, such as a plasmid-like bin. Analyses of these bins revealed various probiotic mechanisms and *bai* operon as probiotic *Ruminococcaceae*’s potential marker. In different health–disease cohorts, these bins were more common in healthy individuals, signifying their probiotic role, but relevant health predictions based on the abundance profiles of these bins faced cross-disease challenges. To better understand the heterogeneous nature of probiotics, we used metaProbiotics to construct a comprehensive probiotic genome set from global gut metagenomic data. Module analysis of this set shows that diseased individuals often lack certain probiotic gene modules, with significant variation of the missing modules across different diseases. Additionally, different gene modules on the same probiotic have heterogeneous effects on various diseases. We thus believe that gene function integrity of the probiotic community is more crucial in maintaining gut homeostasis than merely increasing specific gene abundance, and adding probiotics indiscriminately might not boost health. We expect that the innovative language model–based metaProbiotics tool will promote novel probiotic discovery using large-scale metagenomic data and facilitate systematic research on bacterial probiotic effects. The metaProbiotics program can be freely downloaded at https://github.com/zhenchengfang/metaProbiotics.

## INTRODUCTION

As defined by the World Health Organization (WHO), probiotics are live microorganisms that confer a health benefit on the host when administered in adequate amounts [1]. Some relatively well-studied bacteria have shown multiple distinct probiotic properties, such as protecting intestinal barriers [2], immunomodulation [3], regulating the gut microbiota [4], metabolic regulation [5] and alleviating mental illness through the gut–brain axis [6]. Owing to the health benefits to the host, an enormous effort has been made to discover novel probiotics and understand their probiotic mechanisms in recent years, and the probiotics market is expected to reach $85.4 billion by 2027 [7].

However, determining whether a microorganism is a probiotic may require a comprehensive assessment based on multiple complex factors and lengthy clinical trials. Currently, many molecular mechanisms of probiotics remain unknown. In particular, many intestinal commensal bacteria are potential candidates for next-generation probiotics (NGPs), but challenges in their cultivation often hinder physiological studies [8]. Clinically, selecting and verifying a probiotic strain typically involves a time-consuming randomized controlled trial (RCT) [9], and only certain common genera, such as *Lactobacillus* (which has been reclassified into 25 genera, namely, the emended genus *Lactobacillus*, *Paralactobacillus* and 23 novel genera [10]) and *Bifidobacterium,* can be used to perform large-scale RCTs due to safety concerns. Perplexingly, conflicting results regarding the probiotic effects of certain bacteria often emerge in different studies and reports [11, 12]. Due to the multifaceted interactions between bacteria and their hosts, without a systematic approach for characterizing probiotics within microbial communities, it is challenging to elucidate their specific roles in maintaining host health.

With the accumulation of vast amounts of metagenomic data, the machine learning approach makes it possible for systematic, large-scale probiotic mining using deoxyribonucleic acid (DNA) data, bypassing the above difficulties. Such techniques can derive decision rules from labeled datasets, enabling effective assessments of new data. Their recent application in microbial genomic annotation tasks [13, 14] and the increasing amount of genomic data on known probiotics [15, 16] highlight the potential of such approaches in the field of probiotic mining.

Sun *et al*. introduced such a related tool, iProbiotics, which utilizes *k*-mer frequencies to characterize complete bacterial genomes and employs the support vector machine algorithm for probiotic identification [17]. While iProbiotics has been validated using complete bacterial genomes, its efficacy in draft genomes from metagenomes remains uncertain. Despite the application of the *k*-mer frequency model in various bioinformatics tasks [18], it primarily captures oligonucleotide occurrence frequencies and may not reflect sequence function. The limitations of *k*-mers include redundancy in conserved regions [19–22], a lack of specificity [23, 24] and high-dimensional feature spaces with high *k* values [18]. Metagenomic sequences are often fragmented due to poor assembly, which can lead to fluctuating *k*-mer frequencies. Recently, natural language processing (NLP) techniques have offered innovative biological sequence representation methods. In such models, oligonucleotides or oligo-amino acids are treated as ‘words’ and DNA or protein sequences as ‘sentences’. Through unsupervised pretraining on large datasets, each word is mapped to a context-based feature vector, potentially offering more informative representations than *k*-mer frequencies. This approach has shown success in tasks such as antimicrobial peptide prediction [25], secreted effector identification [26], microbial gene function interpretation [27] and human-virus–protein interaction prediction [28].

In this study, we developed the metaProbiotics tool, which digitally characterizes DNA sequences in metagenomic bins using word vectors and employs random forests for the identification of bins originating from probiotics. Upon assembling and binning the raw metagenomic data using sequence assembly and binning tools, users can then utilize metaProbiotics to identify probiotics from the binned data. Our aim was to overcome the limitations of traditional methods, such as wet-lab experiments and clinical trials, used for probiotic discovery. This approach offers biologists an efficient, systematic method for characterizing probiotic communities. The metaProbiotics program can be freely downloaded at https://github.com/zhenchengfang/metaProbiotics.

## RESULTS

### The unsupervised pretrained word vector for sequence representation

**Figure 1:**
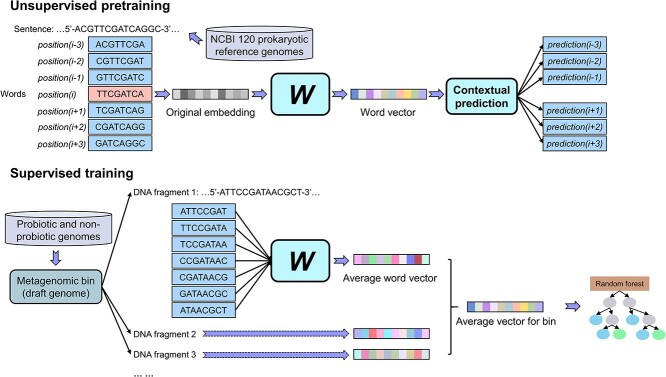
The framework of metaProbiotics. The development of metaProbiotics involved two main steps. The unsupervised pretraining step used the skip-gram algorithm to convert each 8-mer into a word vector language model. Taking the word vector as the mathematical model for the DNA sequences, the supervised training step employed the experimentally validated probiotic and non-probiotic genomic data to construct a random forest for the identification of metagenomic bins of probiotics.

The construction of metaProbiotics primarily involved two steps, as illustrated in Figure 1. During unsupervised pretraining, we employed the skip-gram algorithm, using 120 reference prokaryotic genomes from the National Center for Biotechnology Information (NCBI) (https://ftp.ncbi.nlm.nih.gov/genomes/GENOME_REPORTS/prok_reference_genomes.txt) as the ‘corpus’. Based on the contextual information of nucleotide sequences in DNA, each 8-nucleotide combination was mapped to a word vector with a dimension of 100. For the given DNA sequence(s) from a complete genome, metagenomic fragment or set of fragments from a metagenomic bin, the average word vector of all 8-mers was used to represent the sequence(s).

Using the word vector model, we constructed feature vectors for 237 experimentally validated probiotic genomes from 69 species and 470 non-probiotic genomes from 105 species collected by Sun *et al*. [17] (link: http://bioinfor.imu.edu.cn/iprobiotics/public/Download, hereafter referred to as the Sun dataset).

The traditional *k*-mer model solely quantifies the frequency of various nucleotide strings in DNA, failing to capture the biochemical properties inherent in different nucleotide combinations. In contrast, the word vector model, by analyzing the context of nucleotide strings across diverse sequences, maps contextually similar nucleotide strings to similar spatial coordinates. This spatial representation enables the biochemical characteristics of nucleotide sequences to be more effectively reflected, thereby enhancing the characterization of a species’ biological functions [29]. To compare the advantages of the word vector model with the traditional *k*-mer model, we performed t-distributed Stochastic Neighbor Embedding (tSNE) dimensionality reduction and visual analysis on both the 8-mer’s word vector and 8-mer models for probiotics and non-probiotics, focusing on their spatial distribution characteristics (Figure S1A and C). We found that both models displayed distinct clustering characteristics for probiotics, with approximately four prominent probiotic clusters in the tSNE plots of both the 8-mer and word vectors. However, in the 8-mer tSNE plot, one probiotic cluster was surrounded by non-probiotics and tended to merge with nearby non-probiotics after *k*-means clustering (Figure S1C and D), indicating that the 8-mer model does not perfectly separate probiotics in the coordinate space. By contrast, in the word vector tSNE plot, probiotic clusters were not significantly surrounded by non-probiotics and formed relatively independent groups after *k*-means clustering (Figure S1A and B). We further assessed clustering efficacy using silhouette coefficients, independent of label information, and mutual information, dependent on label information. The silhouette coefficients for each clustered point in the word vector model were significantly higher than those in the 8-mer model (Figure S1E, *P* = 9.42e-19^*^^*^^*^, rank sum test), and the mutual information for the word vector model clustering also surpassed the 8-mer model (Figure S1F), demonstrating the superiority of the word vector model in characterizing the function of probiotics.

**Figure 2:**
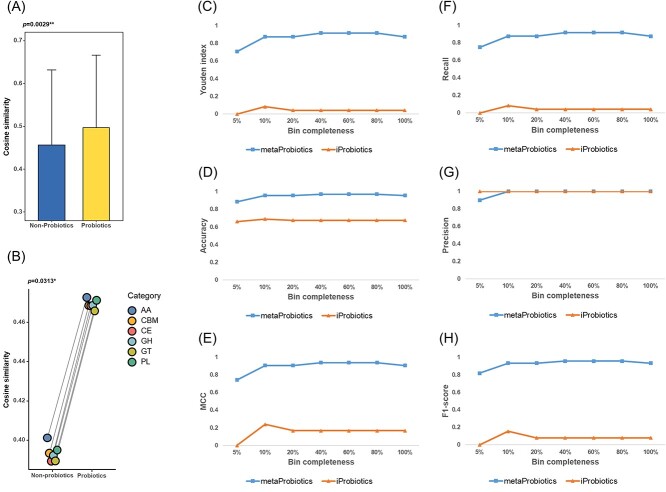
Utilizing cosine similarity to demonstrate the characterization ability of the word vector model for probiotic properties (**A**, **B**) and comparing the performance of metaProbiotics and iProbiotics using simulated metagenomic bin benchmark datasets (**C**–**H**). (A) The average cosine similarity of word vectors between the bacterial genome and genes related to survival and colonization ability, including *clpE*, *dps*, *bsh*, *gadC*, *msrB*, *clpC*, *copA*, *met*, *ispA*, *treC* and *lsp*. The probiotics showed significantly higher cosine similarity than the non-probiotics (*P* = 0.0029^*^^*^, rank sum test). (B) The average cosine similarity of word vectors between the bacterial genome and genes from each category of the CAZy database. The probiotics showed significantly higher cosine similarity than the non-probiotics (*P* = 0.0313^*^, paired rank sum test). (C–H) Performance comparison between metaProbiotics and iProbiotics using the test set bins with different completeness; the (C) Youden index, (D) accuracy, (E) MCC, (F) recall, (G) precision and (H) F1-score were used for the comprehensive evaluation.

We employed cosine similarity from NLP to assess whether word vectors can highlight essential attributes of probiotics. As part of the preclinical evaluation, probiotics should exhibit robust survival and colonization capabilities, particularly within the gastrointestinal tract [30]. We found that the average cosine similarity between the word vectors of probiotics and those of genes associated with bacterial colonization ability (specifically *clpE* and *dps* for bile resistance; *bsh* for bile salt hydrolase; *gadC* for acid resistance; *msrB* for heavy metal exclusion; *clpC* for persistence; *copA* and *met* for competitiveness; *ispA* for adherence; *treC* for growth; and *lsp* for adsorption [17, 31]) was significantly greater than the similarity between these genes and the word vectors of non-probiotics (Figure 2A).

Another pivotal attribute of probiotic microorganisms is their carbohydrate metabolism capacity, including the conversion of specific carbohydrates into propionic, lactic or acetic acid [32–34]. Genes were sourced from the Carbohydrate-Active enZYmes (CAZy) database [35], and the mean word vector for each CAZy class was determined, including Auxiliary Activities (AA), Carbohydrate-Binding Modules (CBM), Carbohydrate Esterases (CE), Glycoside Hydrolases (GH), GlycosylTransferases (GT) and Polysaccharide Lyases (PL). Subsequently, the cosine similarity between probiotics and these CAZy classes as well as between non-probiotics and these classes was computed. Notably, the average cosine similarity of probiotics to each CAZy class was significantly increased compared to that of non-probiotics (Figure 2B).

On the whole, the unsupervised word vector model effectively distinguishes between probiotics and non-probiotics and can highlight related attributes of probiotics.

### Benchmarking the supervised random forest for probiotic mining

In the supervised training phase, a random forest model for probiotic mining was developed using the word vector model. The Sun dataset genomes [17] (link: http://bioinfor.imu.edu.cn/iprobiotics/public/Download) were partitioned into training and test sets at a 9:1 ratio (Table S1). For benchmarking, DNA fragments were randomly extracted from the complete genomes of the Sun dataset to form simulated metagenomic bins. Given the challenges of full genome reconstruction from metagenomes, bins with completeness ranging from 5% to 100% were created, with sequence lengths uniformly distributed between 2000 and 20 000 bp. These bins were mapped to pretrained word vectors. Following 10-fold cross-validation on the training set, the random forest was chosen as the classifier due to its superior accuracy and area under the curve (AUC) among other related machine learning algorithms (Figures S2 and S3).

After the supervised training of the random forest on the training set bins, we evaluated the performance of metaProbiotics against iProbiotics using the benchmark test set bins. The evaluation metrics included the Youden index, accuracy, Matthews correlation coefficient (MCC), recall, precision and F1-score. Across all bin completeness levels, metaProbiotics consistently outperformed iProbiotics (Figure 2 C–H). While the performance of metaProbiotics slightly decreased at 5% completeness, it remained robust otherwise. iProbiotics struggled with fragmented metagenomic data, particularly in the Youden index and MCC, both of which are sensitive to prediction bias. Notably, iProbiotics often misclassified sequences as negative in fragmented data, leading to reduced metric values. Using the *Lactobacillus* genus as an example, we assessed the ability of metaProbiotics to predict probiotics across species within the same genus. The evaluation of *Lactobacillus* bins from the test set revealed an impressive AUC of 94.31% (Figure S4), suggesting that metaProbiotics maintains a robust resolution at the species level.

We further collected draft genomes for *Lactobacillus* and *Bifidobacterium* strains sequenced and submitted to NCBI, including *Lactobacillus paracasei* (recently reclassified as *Lacticaseibacillus paracasei*) and *Bifidobacterium breve*, which are typically considered as probiotics, and *L. iners*, which is typically not considered as probiotic. We found that for draft genomes from *L. iners*, both metaProbiotics and iProbiotics predicted them as non-probiotics. For species commonly classified as probiotics, metaProbiotics was largely able to predict these draft genomes as probiotics, whereas iProbiotics only identified a few high-quality *Lc. paracasei* draft genomes (average contig length > 20 000 bp, N50 > 60 000 bp) as probiotics (Tables S2–S4). This suggests that metaProbiotics has a relatively lower requirement for genome completeness, making it more suitable for mining probiotics in complex metagenomic data. Additionally, through literature research, we collected genomes of six recently isolated and sequenced *Bifidobacterium* strains reported to have probiotic effects [36–38] (see Table S5 for the accessions). We found that metaProbiotics successfully predicted all of them as probiotics, further demonstrating its potential in identifying novel probiotic strains.

These results indicate that metaProbiotics excels in analyzing fragmented and low-integrity metagenomic data, making it appropriate for probiotic mining, particularly for identifying novel NGPs that are challenging to cultivate from metagenomes.

### Application case for testing metaProbiotics with real gut metagenome

Due to the lack of data labels, real metagenomic data are often challenging to use for evaluating tools designed for metagenomic data analysis [39]. To better demonstrate the performance of metaProbiotics in real-world scenarios, we employed gut metagenomes from cohorts subjected to probiotic interventions, in which bins originating from the intervention strains were utilized as labeled data for the quantitative assessment of metaProbiotics.

**Figure 3:**
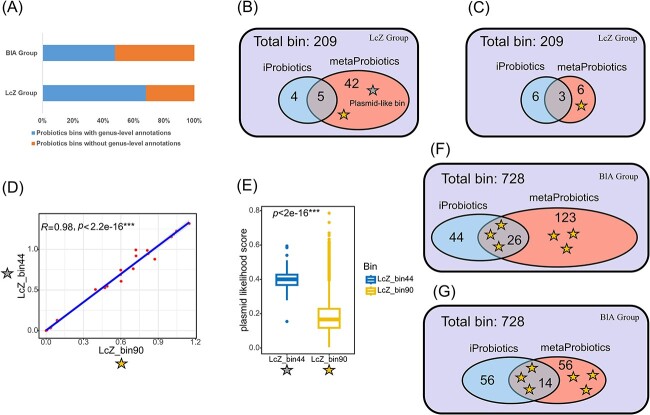
Probiotic mining from healthy human gut samples derived from two probiotic intervention cohorts (LcZ group and BlA group). (**A**) The proportion of probiotic bins identified by metaProbiotics that can be associated at the genus level using the gut reference bacterial genome database (CGR). (**B**) Venn diagram of the probiotic bins identified by the metaProbiotics and iProbiotics tools in the LcZ group. The yellow star (LcZ_bin90) indicates a bin with an ANI higher than 98% against the intervention strains, and the gray star (LcZ_bin44) indicates a plasmid-like bin whose abundance is highly correlated with the intervention strain. (**C**) Venn diagram for the LcZ group. We adjusted the decision threshold to allow metaProbiotics to identify the same number of probiotics as iProbiotics to perform a more quantitative comparison. (**D**) The correlation of abundance between LcZ_bin90 and LcZ_bin44 in samples from different sampling time in the LcZ group. The Spearman correlation index was used. (**E**) The plasmid likelihood score was calculated by PPR-Meta [39] for each contig in LcZ_bin90 and LcZ_bin44. (**F**) Similar to (B), the Venn diagram for the BlA group, yellow stars refer to bins with ANI higher than 98% against the intervention strains. (**G**) Similar to (C), the Venn diagram for the BlA group after adjusting the decision threshold of metaProbiotics.

We employed sample data from two cohorts (see Table S6 for the sample accessions): one was treated with the probiotic strain *Lc. casei* Zhang [40] (a total of 30 samples that were sampled longitudinally, referred to as the LcZ group), and the other was treated with *B. longum* AH1206 [41] (a total of 22 samples from the treatment subgroup, referred to as the BlA group). Sequencing data from both cohorts were subjected to sequence assembly and binning. Ultimately, we obtained 209 bins in the LcZ group and 728 bins in the BlA group, with a notable number of bins exhibiting low completeness (Figures S5 and S6). By calculating the average nucleotide identity (ANI) with genomes in the Culturable Genome Reference (CGR) [42], we annotated the taxonomic units of bins identified by metaProbiotics as originating from probiotics. In both groups, a substantial number of bins could not be assigned to specific genera (Figure 3A), suggesting that a significant portion of the probiotics identified by metaProbiotics either had an unknown taxonomic classification or originated from unculturable genera. This underscores the potential of metaProbiotics to reveal novel probiotics.

We further observed that in both the LcZ and BlA groups, the overlap between probiotics predicted by metaProbiotics and iProbiotics was notably limited. Bins with an ANI greater than 98% to either the *Lc. casei* Zhang or *B. longum* AH1206 genome were considered to have originated from these two strains. Remarkably, metaProbiotics accurately revealed the probiotic property of all these bins. In contrast, iProbiotics struggled to recognize these bins, as evident from the Venn diagram, in which these bins were primarily predicted by metaProbiotics (Figure 3B and F). Given that metaProbiotics predicted more bins than iProbiotics, we adjusted the decision threshold of metaProbiotics to match the number of positive predictions for iProbiotics, ensuring that its broader coverage was not due to leniency. Even so, metaProbiotics still identified all bins closely resembling the intervention bacteria (Figure 3C and G), indicating that the discrepancy in prediction counts between metaProbiotics and iProbiotics was due to the low sensitivity of iProbiotics, rather than an elevated false-positive rate of metaProbiotics. In analyzing the six bins from the BlA group associated with the intervention probiotic strain, we further assessed the quality of these bins and the predictive outcomes of two tools. We found that iProbiotics is particularly sensitive to the length of contigs within a bin and the bin’s overall completeness, identifying only those bins with N50 > 40 000 bp and Completeness >70% as probiotics (Table S7). On the other hand, metaProbiotics demonstrated the ability to identify all bins as probiotics, including those with lower quality, indicating a more robust performance.

In the LcZ group, a bin (bin ID: LcZ_bin44) whose abundance was strictly synchronized with that of the *Lc. casei* Zhang-derived bin (bin ID: LcZ_bin90) was predicted as a probiotic by metaProbiotics (Figure 3B and D). Although its taxonomy was unassigned, it encodes the *Lactobacillaceae*-derived *prtP* gene, a secreted protease that breaks down milk proteins, suggesting a synergistic prebiotic function with *Lc. casei* Zhang. According to PPR-Meta [39], contigs in LcZ_bin44 showed significantly higher plasmid likelihood scores (Figure 3E), implying a plasmid origin of LcZ_bin44 and highlighting metaProbiotics’ ability to detect mobile elements with probiotic functions.

To assess the tool’s generalizability, we focused on the common genera that are not present in the probiotic training set, which have more than one bin in either cohort and at least one bin predicted as probiotics by the metaProbiotics tool. These genera, including *Butyricicoccus*, *Parabacteroides*, *Phocaeicola*, *Agathobaculum*, *Alistipes*, *Collinsella* and *Ruminococcus*, have reported probiotic capacities, including functions such as short-chain fatty acid (SCFA) production, immunomodulation, metabolic regulation and intestinal barrier protection via diverse molecular mechanisms (Table S8).

**Figure 4:**
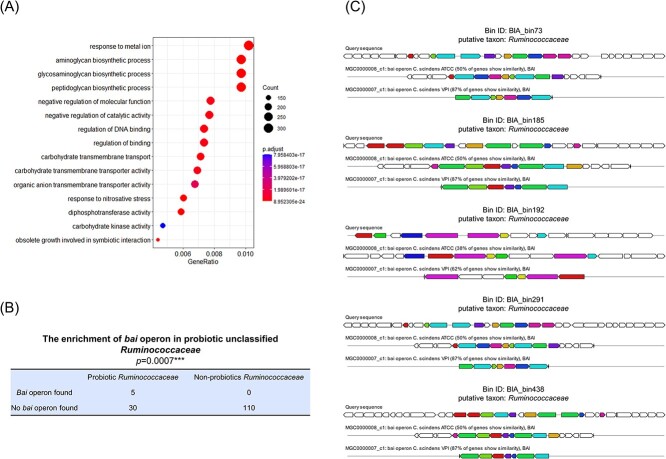
Gene function analysis of the probiotics identified by metaProbiotics. (**A**) GO enrichment analysis of probiotic-encoded genes. (**B**) The enrichment of the *bai* operon in unclassified *Ruminococcaceae* probiotics (*P* = 0.0007^*^^*^^*^, Fisher’s exact test). All five unclassified putative *Ruminococcaceae* bins containing *bai* operons were identified as probiotics, while no *bai* operon was found in non-probiotic *Ruminococcaceae* bins. (**C**) The *bai* operons in *Ruminococcaceae*-related bins. The contigs with *bai* operons and the alignment graph with reference *bai* operons are shown.

Gene function analysis based on Gene Ontology (GO) enrichment indicated that the probiotic bins identified by metaProbiotics encode a variety of genes linked to probiotic mechanisms (Figure 4A). Notably, in addition to genes involved in carbohydrate metabolism, which are traditionally deemed essential for probiotics, there were genes tied to metal–iron responses. This finding was in accord with observations that certain probiotics can regulate the harmful accumulation and toxicity of heavy metals in humans [43, 44]. Furthermore, these identified probiotics show abundant genes associated with the synthesis of substances exhibiting probiotic effects, including glycosaminoglycan, which is known to protect against diseases such as ankylosing spondylitis [45] and rheumatoid arthritis [46].

Interestingly, focusing on the bins associated with the family of unclassified *Ruminococcaceae*, we found that all five *Ruminococcaceae*-related bins containing the *bai* operon were identified as probiotics by metaProbiotics, whereas no *bai* operon was found in non-probiotics *Ruminococcaceae* bins (Figure 4B and C). Our prior research indicated that certain *Ruminococcaceae* can ameliorate chronic insomnia and cardiometabolic diseases via the microbiota–bile acid axis [47]. Thus, based on our findings, we speculate that promoting bile acid metabolism is an important factor in the prebiotic functions of some *Ruminococcaceae* and that genes related to bile acid metabolism, such as the *bai* operon, are the key genes responsible for the probiotic functions of these bacteria.

We further evaluated the capability of metaProbiotics to identify probiotics from metagenomic binning data derived from various sources, thereby demonstrating the tool’s universality. We utilized data from Huang *et al.* [48], in which they conducted interventions on human and mice populations using the probiotic strain *Lactiplantibacillus plantarum* HNU082. We downloaded metagenomic data from these human and mice subjects and processed it similarly to the LcZ and BlA groups. This process yielded metagenomic bins from different populations, which were then predicted using metaProbiotics and iProbiotics. The results indicate that metaProbiotics consistently identified bins derived from the intervention strain as probiotics, outperforming iProbiotics (Figure S7). This suggests that the efficacy of metaProbiotics in identifying probiotics is not confined to specific types of metagenomic data, thus indicating a broad range of applicability.

### Consistency and heterogeneity in probiotic abundance profiles across healthy and diseased individuals

**Figure 5:**
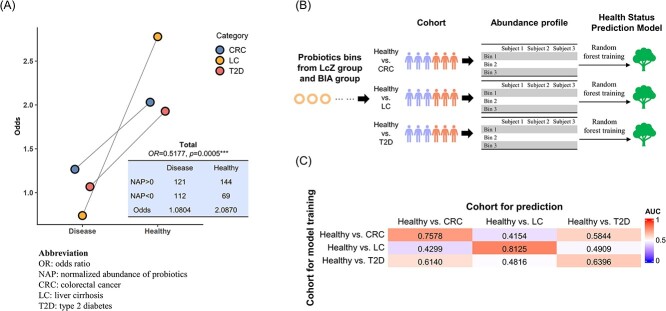
The abundance profile of the identified probiotic bins among cohorts of individuals with different conditions, including healthy, CRC, LC and T2D. (**A**) Comparison of the overall NAP between healthy individuals and those with specific diseases across different cohorts. The high abundance of probiotics (sum of NAP exceeding 0) was designated as the exposure factor, based on which the odds values in each cohort were determined. The relationship between probiotic abundance and health status is depicted in the light-blue box. This demonstrates that probiotics identified by metaProbiotics act as a protective factor across different diseases (OR = 0.5177, *P* = 0.0005^*^^*^^*^, one-sided Fisher’s exact test). (**B**) In each cohort, a health status prediction model based on a random forest was trained to identify human health status using the abundance profiles of probiotic bins in each healthy individual and each individual with a specific disease. (**C**) For each model in (B), we predicted the health status of every individual in each cohort with a specific disease and calculated the predictive AUC. When the cohort used for training was the same as the cohort used for prediction, we employed a leave-one-out approach to evaluate the AUC. We observed that when the prediction cohort was different from the training cohort, there was a significant decline in the AUC, particularly for cross-cohort predictions between CRC and LC.

We assessed the abundance of probiotic bins identified from the above application cases by metaProbiotics in the gut metagenome of individuals across various cohorts. Each cohort consisted of samples from both healthy individuals and individuals with specific diseases, including type 2 diabetes (T2D) [49], liver cirrhosis (LC) [50] and colorectal cancer (CRC) [51–54] (see Table S9 for the samples’ accessions). We found that in cohorts of different disease types, the proportion of healthy individuals with a total normalized abundance of probiotics (NAP) greater than 0 consistently exceeded that of disease patients, indicating a higher odds value for health (Figure 5A: bins present in all individuals in a certain cohort were used for statistic; Figure S8: all bins were used). When considering a total NAP >0 as an exposure factor, these probiotic bins could serve as protective factors against diverse diseases (OR = 0.5177, *P* = 0.0005^*^^*^^*^, the light-blue boxes in Figure 5A), underscoring their consistent roles across various diseases.

However, we also observed that among cohorts of different disease types, the distribution patterns of the abundance of these probiotics between healthy individuals and disease patients might show significant heterogeneity. Following the procedure depicted in Figure 5B, we attempted to train a health status prediction model based on random forests using the abundance features of these probiotic bins for each disease type. However, these models struggled with cross-disease predictions; when they were trained on one disease type and tested on another, the predictive AUC notably decreased. This decline was especially evident for CRC and LC, where the AUC for cross-disease predictions dropped below 50%. This suggests that probiotics may exhibit considerable variability in their protective roles against different diseases. We further explored the possibility of identifying a core subset of probiotics that play a significant role in health status prediction across different diseases, potentially aiding in future cross-disease universal predictions. Using feature importance calculation based on out-of-bag (OOB) in random forests, we pinpointed the top 20 bins of highest importance in health status prediction models developed using various disease cohorts. These bins were then examined for their shared presence across different models using a Venn diagram (Figure S9). The results revealed that we could not identify any bins that were crucial for health status prediction in all three diseases analyzed. Additionally, there were only a few bins that played a significant role in at least two diseases. This further suggests that the heterogeneous expression of probiotics across different diseases is quite pronounced, and identifying a group of bacterial species beneficial for all types of diseases remains challenging.

We further collected cohorts from two other disease types, including irritable bowel syndrome (IBS) and Crohn’s disease (CD) [55, 56] (see Table S10 for the samples’ accessions). After undergoing similar analyses, we also observed patterns of consistency and heterogeneity in probiotics. Specifically, the overall abundance of probiotics can be considered a protective factor across various diseases, but health status prediction models constructed using these probiotic abundance features struggled to achieve favorable outcomes in cross-disease predictions (Figure S10). We further investigated whether expanding the range of diseases in the training set of the health prediction model could enhance its cross-disease predictive ability. We merged the cohorts of CRC, LC and T2D (i.e. combining healthy individuals from all cohorts into one category and those with any of the diseases into another) for training the health status prediction model. Subsequently, we applied the trained model to predict health status in IBS and CD cohorts. We found that increasing the variety of diseases in the training set did not significantly improve the model’s ability of cross-disease predictions (Figure S10B). The variation of probiotics across different diseases continues to impede the development of a universal predictive model.

### Revealing the heterogeneity of probiotic gene modules across diseases using the global gut probiotic set created by metaProbiotics

**Figure 6:**
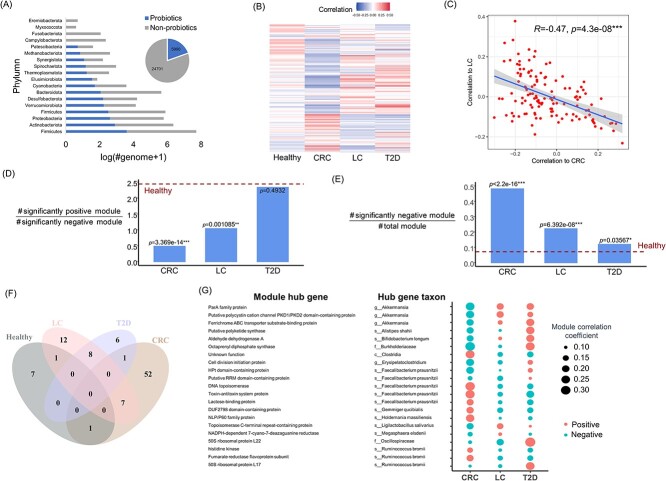
Using metaProbiotics to construct a comprehensive gut probiotic set and revealing the abundance patterns of gene modules within these probiotic communities in healthy individuals and in different disease groups (including CRC, LC and T2D) through WGCNA. (**A**) Utilizing metaProbiotics to construct a dataset resource for human gut probiotics from the 30 691 metagenome-assembled genomes in the HumGut database. The pie chart displays the number of probiotic genomes sourced from HumGut, and the bar chart shows the phylum distribution of the identified probiotics. (**B**) Calculating the abundance of genes encoded by probiotics in (A) among healthy individuals and specific disease groups (CRC, LC and T2D). Using WGCNA, these genes were divided into different gene modules. Each row in the heatmap represents a gene module; the color of the module indicates its correlation with the respective phenotype. (**C**). Illustrating the correlation of each probiotic gene module with CRC and LC diseases, identifying a contrasting trend in the module’s correlation with the two conditions. The Spearman correlation index was used. (**D**) Determining the ratio of significantly positively correlated module counts to significantly negatively correlated module counts for each disease and providing a reference value for this ratio in the healthy population (indicated by the red dashed line). The *P*-value was obtained through a one-sided binomial test based on the proportion of positive modules to the total statistical modules. (**E**) Calculating the ratio of significantly negatively correlated module counts to the total module count for each disease, providing a reference value in the healthy population (red dashed line). The *P*-value was obtained through a one-sided binomial test. (**F**) Venn diagram displaying the modules with significant negative correlations across different phenotypes. (**G**) Showcasing hub gene information for modules showing significant correlation (*P* < 0.05, including both positive and negative correlations) across the three diseases. This includes the proteins encoded by the hub genes, the taxon of the genome containing the hub gene and a bubble chart indicating the magnitude of the correlation of the corresponding gene module in different diseases.

To elucidate the heterogeneous behavior of probiotics in various diseases based on a comprehensive view of the gut probiotic community, we utilized metaProbiotics to identify probiotics from 30 691 metagenome-assembled genomes (MAGs) sourced from over 5700 global healthy gut samples in the HumGut database [57], ultimately yielding 5990 probiotics spanning different phyla (Figure 6A). This genomic set significantly expands the species coverage of the current probiotic databases. The information of this probiotic is provided in the ‘probiotics_set.csv’ file in the GitHub site https://github.com/zhenchengfang/metaProbiotics/releases/tag/Globle_gut_probiotics_set.

Utilizing the gut metagenomic data from the aforementioned individuals who were healthy or had one of the CRC, LC and T2D, we calculated the abundance of each gene from the 5990 probiotics constructed by metaProbiotics across different phenotypic groups. These probiotic genes were then categorized into gene modules using weighted correlation network analysis (WGCNA) (Figure 6B). As shown in Figure 6B, certain gene modules in the healthy condition exhibited a degree of enrichment (positively correlated with the phenotype), with no substantial module depletion (negatively correlated), indicating a relatively complete composition of probiotic gene modules in the healthy state. In contrast, within various disease conditions, while there was enrichment of gene modules, a conspicuous depletion of gene modules was also evident. This suggests that the enrichment of certain probiotic gene modules may not necessarily promote host health, and the onset of diseases often coincides with the loss of gene modules.

Concurrently, we observed substantial variations in the types of modules that were enriched or depleted across different disease states. For instance, modules that were positively correlated with CRC appeared to be negatively correlated in LC and vice versa, indicating a pronounced antagonistic relationship (Figure 6C). This could also explain the AUC value below 0.5 observed in Figure 5C when making cross-cohort health status predictions for CRC and LC. Furthermore, we examined the ratio of significantly positively correlated modules to negatively correlated modules (*P* < 0.05) in each condition. We found that this ratio was consistently lower in various disease states compared to the healthy state, although the difference was not always statistically significant (Figure 6D). Additionally, we assessed the proportion of significantly negatively correlated (*P* < 0.05) modules among the total modules in each state. We observed that this proportion was significantly higher in disease states than in the healthy state (Figure 6E), with considerable variation in the missing modules across different conditions (Figure 6F). These observations suggest that the integrity of gene functions within the probiotic community is more crucial for maintaining gut homeostasis than merely increasing the abundance of specific functional genes. The same probiotic gene module exhibits heterogeneity across different diseases, making it challenging to determine the host’s health status based solely on the enrichment or absence of specific probiotics.

Building on this, we further identified modules that were significantly correlated (both positively and negatively) across CRC, LC and T2D. We pinpointed the hub genes of these modules (a total of 21 hub genes) and documented the taxonomic information of the genomes containing these hub genes as well as their module correlations across various conditions (Figure 6G). We again observed that the probiotic effects on diseases exhibited heterogeneity. No taxon associated with any of the hub genes was found to have probiotic benefits across all analyzed diseases. For instance, the probiotic *Akkermansia* exhibited beneficial effects (negatively correlated) for CRC but not for LC or T2D. Moreover, different genes from a specific probiotic species can be categorized into different gene modules of the community, and a single module can have varied probiotic effects across diseases. For example, *Faecalibacterium prausnitzii* had five genes serving as hub genes for five modules. Two of these hub genes (HPt domain–containing protein and putative RRM domain-containing protein) were beneficial for CRC and LC but not for T2D, while the other three exhibited the opposite effect, offering no protective benefits for CRC but being beneficial for LC and T2D. Similarly, *Ruminococcus bromii* had three genes identified as hub genes for the three modules. Two of these modules were beneficial for LC and T2D but not for CRC, while the other module was beneficial for CRC and LC but not for T2D.

Overall, utilizing the comprehensive gut probiotic set constructed by metaProbiotics, we elucidated the significance of probiotic gene functional integrity in maintaining host health, taking CRC, LC and T2D as examples. We also highlighted the heterogeneous characteristics of probiotics across different diseases. We believe that these findings offer insights for the clinical application of probiotics. Specifically, not all functional modules of probiotics may be protective against every disease. In interventions involving probiotics, the integrity of microbial functions should be prioritized, as an excessive increase in the abundance of specific probiotics might, in fact, produce undesirable outcomes.

## DISCUSSION

The language model of metaProbiotics considers eight adjacent bases as a ‘word’ to construct word vectors. However, in the genome, genes or domains are often the basic units of function. Some studies have also considered genes as ‘words’ for modeling [58]. metaProbiotics is mainly aimed at next-generation sequencing data of the metagenome. The length of contigs in these data is often short, while a complete metabolic gene cluster generally contains at least five genes. If using a gene as a ‘word’, it is unlikely that a contig obtained from next-generation sequencing will contain a complete ‘sentence’, which does not allow a full demonstration of the advantages of the language model. Therefore, we chose the string of bases instead of the entire gene as a word unit to better capture the different motifs in the DNA sequence. By combining ultrahigh-depth next-generation sequencing and third-generation sequencing, it is now possible to reconstruct a complete bacterial genome from metagenomic data instead of fragmented draft genomes [59]. Therefore, in the future, we will further combine the data characteristics of different sequencing technologies to build genome language models from different dimensions and improve the algorithm’s discrimination of probiotic genomes.

metaProbiotics cannot directly utilize raw sequencing reads to identify probiotics within a metagenome. Therefore, the ability to reconstruct the draft genome of probiotics from the metagenome through sequence assembly and binning is a prerequisite for the successful identification of probiotics by metaProbiotics. Numerous assembly and binning tools have been published, with common assembly tools including MegaHit [60], IDBA-UD [61], metaSPAdes [62] and MetaVelvet [63] and common binning tools including MetaBAT2 [64], MaxBin [65] and CONCOCT [66]. However, studies have shown that different tools exhibit varying preferences for samples with distinct microbial community structures [67] (such as species abundance and diversity), leading to significant variations in the results obtained from the same sample by different tools. These discrepancies can introduce biases in metaProbiotics’ predictions. For instance, when using binning tools, we attempted binning on metagenomic data from six mouse samples [68] (accession: PRJNA293015) using both MetaBAT2 and MaxBin, resulting in 239 and 448 bins, respectively, a substantial difference. Without prior knowledge of the microbiota, it is challenging to determine which result is more accurate. To integrate the strengths of different tools, comprehensive workflows such as MetaWRAP [69] and Metaphor [70] have been developed. These tools often improve accuracy compared to individual tools but at the cost of significantly longer run times, making them less suitable for large-scale data processing. We recommend that readers choose tools flexibly based on their needs. For instance, if a user’s primary interest lies in focusing on the dominant species in a microbial community rather than the diversity of species, metaSPAdes can be utilized for sequence assembly. This tool is capable of generating longer contigs but is less sensitive to species diversity [67], when dealing with samples exhibiting high species diversity and the presence of low-abundance species, there may be a loss of information. For smaller sample sizes, comprehensive but time-consuming workflows like MetaWRAP can be used to enhance the accuracy of downstream metaProbiotics. For detailed information on common sequencing assembly and binning tools, readers may refer to reviews by Yang *et al* [71]. Moreover, if researchers aim to obtain high-quality metagenomic binning data, they may opt to use Hi-C sequencing data. This sequencing technique involves crosslinking DNA from the same bacterial cell prior to sequencing, allowing for more accurate binning of DNA sequences post-sequencing, utilizing the crosslinking information [71, 72]. However, compared to standard metagenomic sequencing, Hi-C sequencing is more costly, and researchers should select the data type based on their specific needs.

In some cases, binning software may group different strains with high sequence similarity into the same bin. For instance, in Table S7, we identified a bin with a contamination level of 281.82%. This bin likely contains sequences from both probiotic strains and other bacterial strains. However, the current design of metaProbiotics does not allow for further differentiation between components from probiotics and other strains within a bin. On one hand, as discussed earlier, we can use a combination of different binning tools to further optimize the binning quality and obtain purer probiotic sequences. On the other hand, analysis in this paper suggests that probiotics exhibit relatively distinct modular characteristics in their genes when exerting probiotic effects. In subsequent research, it is necessary to further expand the capabilities of our tool to include enhanced modeling and characterization of various gene modules within the genome, along with providing more comprehensive functional annotations for these modules. This will enable metaProbiotics to answer the question ‘which gene modules in a bin are responsible for specific probiotic functions?’ This approach could not only help researchers extract core functional modules from contaminated data for in-depth mechanistic studies but also provide guidance for the rational use of probiotics in clinical settings.

One of the significant findings of this work is that probiotics exhibit heterogeneous behavior across different disease types. Predicting this heterogeneity based on the probiotic community structure and host factors and establishing a robust health state prediction model to enhance personalized probiotic interventions are crucial for future research. In fact, the probiotic properties of certain bacteria might be influenced by various factors, such as the abundance profile of other bacteria, environmental conditions and the host’s diet. While metaProbiotics can accurately capture vital information related to probiotic functionality from primary sequences using language models, it does not consider other external factors in the probiotic discrimination process. This limitation could hinder the application of metaProbiotics in personalized disease prevention and treatment. In recent years, numerous bioinformatics tools have been developed for predicting the microbiome–drugs interaction, as well as the microbiome–diet interaction [73, 74]. These tools are immensely helpful in further analyzing the heterogeneous effects of probiotics in different individuals. Users can integrate them with their specific needs for further analysis of identified probiotics, serving as a complement to metaProbiotics. Since these tools consider a broader range of factors, they may require more diverse data type inputs, such as nutrient intake matrices [74]. Therefore, it is advisable to have data from specifically designed experiments before utilizing these tools. In our future work, we plan to utilize large-scale population cohort data, gather comprehensive host information and leverage these multimodal data to establish mathematical models for bacterial strains. These models incorporate diverse information, including genomic data, environmental factors and host details, to develop a more holistic discrimination algorithm. We aspire to ultimately guide personalized medicine tailored to individual differences through big data–driven AI algorithms. This includes personalized probiotic interventions based on individual dietary habits and lifestyle information, as well as customized dietary and medication recommendations based on the characteristics of an individual’s probiotic microbiota.

In conclusion, leveraging artificial intelligence language models, we introduced a novel computational approach to identify probiotics from metagenomic bin data. This method facilitates the discovery of novel probiotics and offers a fresh perspective, systematically elucidating the characteristics and heterogeneous manifestations of probiotic communities in maintaining host health.

## METHODS

### Pretraining of word vectors

We downloaded 120 representative prokaryotic genomes from NCBI (https://ftp.ncbi.nlm.nih.gov/genomes/GENOME_REPORTS/). These 120 bacteria contained a total of 240 DNA sequences derived from bacterial chromosomes and plasmids, and we also extracted the complementary sequence for each DNA sequence, doubling the number of DNA sequences to 480. These 480 sequences were then used for the pretraining of word vectors. In the metaProbiotics language model, each set of *k* adjacent bases is treated as a word, where *k* can be fixed or can vary during processing. According to Sun *et al*. [17], oligonucleotide markers distinguishing probiotics from non-probiotics are predominantly found in oligonucleotides with higher *k* values (*k* > 5), where the top four oligonucleotides with the highest importance scores are 8-mers. Therefore, a fixed *k* of 8 was used to train the word vector. All overlapping 8-mers were extracted from the 480 sequences, and each 8-mer combination was embedded into an initialized numeric vector. Using the skip-gram-based dna2vec algorithm [75], a neural network with one hidden layer containing 100 nodes was employed for word vector training. During training, the neural network takes an 8-mer string of bases from the DNA sequence as input and predicts 10 adjacent 8-mer strings both upstream and downstream of the initial 8-mer sequence. Assuming that *k*-mers with similar biological properties will have similar sequence contexts, these *k*-mers are likely to produce similar outputs in the final layer. Consequently, during unsupervised training, *k*-mers with analogous biological properties are mapped to similar feature vectors in the hidden layer. The feature vector from the 100-node hidden layer then serves as the word vector embedding for the corresponding 8-mer. Relevant parameters of the dna2vec algorithm during training were set as follows: ‘k=8, vec-dim=100, epoch=10, context=10’. Negative sampling was employed to enhance the efficiency of the standard softmax function. In subsequent algorithm construction and application steps, for any given DNA sequences (whether a single sequence or multiple sequences from a bin), we employ JellyFish [76] (v2.3.0) to obtain all 8-mer strings. All corresponding word vectors of these 8-mer strings are summed, and each feature of the word vector is averaged. Consequently, any given DNA sequence, or multiple DNA fragments within a bin, can be represented by a word vector with a dimensionality of 100. Ultimately, this average word vector represents the word vector for the given DNA sequences.

### Data set construction of probiotics and non-probiotics

We downloaded the experimentally verified probiotic and non-probiotics genomes from Sun *et al.* [17] at http://bioinfor.imu.edu.cn/iprobiotics/public/Download. These probiotics and non-probiotics were randomly divided into a training set and a test set at a 9:1 ratio. The training set was further partitioned into 10 equal parts for 10-fold cross-validation. For each genome in the dataset, the ID information, category and the groups into which it was randomly divided are detailed in Table S1. We then randomly extracted sequence fragments from each genome to construct simulated metagenomic binning data, which were used as a benchmark dataset for the development of metaProbiotics. The lengths of these randomly extracted sequences follow a uniform distribution between 2000 and 20 000 bp. Sequences are extracted with equal probability from either the positive strand or the complementary strand of the genome. In the training set, each genome yielded 20 simulated bins with 5% completeness, 10 bins with 10% completeness, 5 bins with 20% completeness, 3 bins with 40% completeness, 2 bins each with 60% and 80% completeness and 1 bin with 100% completeness. In the test set, each genome produced one bin for each completeness level: 5%, 10%, 20%, 40%, 60%, 80% and 100%.

### Cosine similarity calculation

For a given bacterial genome word vector (B) or gene word factor (g), the cosine similarity is defined as $\frac{1}{2}\left(\frac{\mathbf{B}\bullet \mathbf{g}}{\left|\mathbf{B}\right|\left|\mathbf{g}\right|}+1\right)$. Before calculating the similarity, each element of the word vector will be centered so that it has a zero mean. When calculating the cosine similarity between the genome and colonization-related genes, we collected the base sequences of related genes from NCBI by a keyword search. For example, for the *bsh* gene, we used keywords ‘(bsh[Gene Name]) AND “bacteria”[Organism]’ for searches. Each gene had multiple sequence records, and the average word vector of each record was used as the representative word vector of this gene. For each genome, we calculated its cosine similarity with the representative word vector of each gene and obtained the average cosine similarity, which was used as a measure of similarity related to colonization ability between the genome and genes. When calculating the cosine similarity between the genome and the genes from the CAZy database, the base sequences of genes in the database were downloaded from the dbCAN2 website [77] and then clustered with 90% similarity using the *cd-hit-est* module of CD-HIT [78] (v4.8.1, parameter settings: -c 0.90, -n 8 and -d 100), and the word vector of the representing sequence was calculated. For the genes of each category in CAZy, we took the average word vectors of these genes as the representative word vectors of this category and calculated their average cosine similarity with the word vectors of each probiotic and non-probiotic genome.

### Evaluation of the tools using a simulated benchmark dataset

Both iProbiotics and metaProbiotics can assign scores to input sequences ranging from 0 to 1. A threshold of 0.5 is used for discrimination: sequences with scores above 0.5 are classified as positive samples (probiotics), while those below 0.5 are deemed negative samples (non-probiotics). metaProbiotics can score either a single sequence or multiple sequences from a bin collectively. In contrast, iProbiotics can only evaluate individual sequences sequentially. Given our focus on metagenomic data analysis, where DNA from a bacterial bin often appears as multiple sequence fragments, if multiple sequences from a specific bin are input into iProbiotics, we use the average score of these sequences as the bin’s score. Furthermore, metagenomic sequences vary in length, with longer sequences typically yielding more reliable predictions than shorter ones. Thus, when computing the average score of iProbiotics, we weight each sequence’s score by its length to obtain a weighted average score, which serves as the iProbiotics identification result. When assessing tools based on benchmark datasets from simulated metagenomics, we train metaProbiotics using a training set data and test it on test set data. For real sample applications and released tool versions, we train metaProbiotics using all available data.

During the benchmarking process, the criteria of the Youden index, accuracy, MCC, recall, precision and F1-score are defined as follows:


$$ Youden\ index= Sensitivity+ Specificity-1 $$

$$ Accuracy=\frac{TP+ TN}{TP+ TN+ FN+ FP} $$

$$ MCC=\frac{TP\times TN- FP\times FN}{\sqrt{\left( TP+ FP\right)\left( TP+ FN\right)\left( TN+ FP\right)\left( TN+ FN\right)}} $$

$$ Recall=\frac{TP}{TP+ FN} $$

$$ Precision=\frac{TP}{TP+ FP} $$

$$ F1- score=\frac{2\times recall\times precision}{recall+ precision} $$

where $Sensitivity=\frac{TP}{TP+ FN}$, $Specificity=\frac{TN}{TN+ FP}$, and *TP*, *TN*, *FN* and *FP* refer to the number of true positives, true negatives, false negatives and false positives, respectively.

### Data processing of metagenomic samples from the LcZ group and BlA group

We obtained the sequencing data from the SRA database according to the accessions of the samples. We used fastp [79] for quality control, MegaHit [60] (v1.2.9) for sequence assembly (using default parameters) and then the built-in MetaBAT2 [64] in MetaWRAP [69] (docker version) to bin the contigs assembled ​​from the LcZ group and BlA group (using default parameters). The completeness of each bin was evaluated by CheckM [80] (v1.0.12). To identify the bins associated with the intervening strain, we used fastaANI [81] (v1.33) to calculate the ANI between each bin and the intervening strain genome (*Lc. casei* Zhang: NC_014334.2; *B. longum* AH1206: NZ_CP016019.1) (parameter setting: --fragLen 1000), and bins with an ANI higher than 98% were regarded as bins derived from the intervening strain. To infer the associated taxon of each bin using the CGR database, we used fastANI to calculate the ANI between each bin and each reference genome and used 80% as the threshold for genus determination. If a bin is linked to multiple taxa at the same time, we think that this bin may be mixed with genomes from different bacteria, and this bin will be assigned to multiple taxa at the same time. To identify bins associated with the family of unclassified *Ruminococcaceae*, we searched for bins with an ANI greater than 75% compared to the reference unclassified *Ruminococcaceae* genomes. The ANI thresholds for the genus and family levels were chosen based on the report of Zhang *et al*. [82], and the threshold was slightly lowered considering that the metagenomic bins usually do not contain a complete and pollution-free genome. In the LcZ group, we also used the *quant_bins* module (default parameters) in MetaWRAP to calculate the abundance of each bin. We used eggNOG-mapper [83] for gene function annotation, took all gene GO terms as the background gene set and the probiotic gene GO terms as the target objects and then conducted GO enrichment analysis using the *enricher* function in clusterProfiler [84] (parameter setting: pAdjustMethod = ‘BH’, pvalueCutoff = 0.05, qvalueCutoff = 0.2). The gutSMASH [85] was used to identify the *bai* operon over each bin.

### Abundance profiles of probiotics identified from test populations in cohorts with various disease types

The gut metagenomic reads of cohorts with different disease types (CRC, LC and T2D) were downloaded according to their accession. Both the LC and T2D types contained one cohort with healthy individuals and disease patients, while the CRC type contained three cohorts from different regions (Table S9). The reads passed quality control in T2D cohort was obtained from Feng *et al*. [86], and we used fastp for quality control of other cohorts. We used the *quant_bins* module in MetaWRAP to calculate the abundance of the identified probiotics from the LcZ and BlA groups in each cohort. During the use of MetaWRAP, we used the -b parameter to specify the probiotic bins from the LcZ group and the BlA group and did not specify the -a parameter. To calculate the odds value of each cohort, the abundance of bins was *z* score–normalized; therefore, the positive or negative value indicated the upregulation or downregulation of the bin abundance. When constructing the cross-cohort healthy prediction model, the original abundance value of all probiotic bins served as the random forest input so that the model could perform prediction for a single sample independently.

### Gene modules analysis based on WGCNA

We used Prodigal [87] (v2.6.3) to extract genes from the 5990 predicted probiotic genomes from the global gut metagenome, and we used CD-HIT to reduce redundancy under a 90% similarity threshold. The abundance of each probiotic gene in each individual included in Table S9 was calculated using the *quant* module of salmon [88] (docker version, parameters setting: -l A). Considering that the abundance profile of genes across samples was sparse, we extracted the top 20 000 genes by calculating the total abundance among all the samples to perform the downstream analysis. The gene abundance matrix for the healthy condition and three types of disease conditions was then used for co-expression network analysis using the R package WGCNA [89] (v1.72.1). Then, the network was constructed by the following steps with specific functions: (1) choose a set of powers to analyze the network topology using the *pickSoftThreshold* function (parameters: powerVector = c(1:30), verbose = 5) and then choose 5 as the best power value; (2) use the *adjacency* function to transform the similarity matrix into an adjacency matrix (parameters: power = 5); (3) use the *TOMsimilarity* function to transform the adjacency matrix into a topological overlap matrix (TOM) and use the default parameters; and (4) use the *cutreeDynamic* function to identify modules, based on the dynamic tree cut method (parameters: deepSplit = 2, minClusterSize = 50, pamRespectsDendro = F). The genes were first hierarchically clustered using the *hclust* function (parameters: method = ‘average’) and then transferred to the *cutreeDynamic* function; (5) use the *moduleEigengenes* function to obtain the eigengene in each module (default parameters); (6) use the *mergeCloseModules* function to merge similar gene modules (parameters: cutHeight = 0.25, verbose = 3); and (7) use the *cor* function to calculate the correlation between each module and its phenotype and the *corPvalueStudent* function to calculate the corresponding *p value*. The hub genes are obtained using *chooseTopHublnEachModule* function. All the hub genes were submitted to the UniProt online blast server (https://www.uniprot.org/blast) for function annotation. Based on the UniProt output file, the function of the protein with the highest match will be used for hub gene annotation. If the top-matching protein lacks functional annotation, the highest-matching protein with functional information will be used, prefixed with ‘putative’.

**Key Points**

- The metaProbiotics tool uses the word vector language model to characterize deoxyribonucleic acid sequences and employs the random forest algorithm to rapidly identify the probiotic-derived bins from metagenomic data, demonstrating superior performance in simulated benchmark datasets.
- Testing on gut metagenomes from probiotic-treated individuals, metaProbiotics can better identify the bins with probioticity, including a plasmid-like bin showing synergistic prebiotic function with the intervention strain.
- Gene function analysis revealed multiple probiotic mechanisms of the probiotic bins identified by metaProbiotics and *bai* operon is a potential key marker of some probiotically active *Ruminococcaceae*.
- Probiotic bins identified by metaProbiotics could serve as protective factors against diverse diseases, signifying their probiotic role.
- Large-scale probiotics analysis with metaProbiotics shows that gene function integrity of the probiotic community is more crucial in maintaining gut homeostasis than merely increasing specific gene abundance, owning to the fact that different gene modules on the same probiotic have heterogeneous effects on various diseases; adding probiotics indiscriminately might not boost health.

## Supplementary Material

cover_letter_bbae085

Supplementary_information_1_bbae085

Supplementary_information_2_bbae085

## Data Availability

The list of 120 reference prokaryotic genomes used for language model training can be downloaded from NCBI (link: https://ftp.ncbi.nlm.nih.gov/genomes/GENOME_REPORTS/prok_reference_genomes.txt). The list of the probiotic and non-probiotics genomes used for metaProbiotics construction can be downloaded from Sun *et al*. [17] (link: http://bioinfor.imu.edu.cn/iprobiotics/public/Download), and the organization of these genomes is provided in Table S1. The accession list of metagenomic sequencing data for the LcZ and BlA groups is provided in Table S6. The accession list of the metagenomic sequencing data for cohorts with healthy individuals and different disease types is provided in Table S9. The accession list of the probiotic set constructed by metaProbiotics using the global gut metagenome is provided in the ‘probiotics_set.csv’ file in https://github.com/zhenchengfang/metaProbiotics/releases/tag/Globle_gut_probiotics_set.

## Author Biographies

**Shufang Wu** is a post-doctoral researcher in the Department of Laboratory Medicine at Zhujiang Hospital, Southern Medical University. Her research interest includes bioinformatics and microbial genomics.

**Tao Feng** is a doctor student in the Department of Laboratory Medicine at Zhujiang Hospital, Southern Medical University.

**Waijiao Tang** is a pharmacist in the Department of Laboratory Medicine at Zhujiang Hospital, Southern Medical University.

**Cancan Qi** is a post-doctoral researcher in the Department of Laboratory Medicine at Zhujiang Hospital, Southern Medical University.

**Jie Gao** is a professor in the Department of Gastroenterology at The Second Affiliated Hospital of Guangzhou Medical University.

**Xiaolong He** is a professor in the Department of Laboratory Medicine at Zhujiang Hospital, Southern Medical University.

**Jiaxuan Wang** is an assistant researcher in the Department of Laboratory Medicine at Zhujiang Hospital, Southern Medical University.

**Hongwei Zhou** is a professor in the Department of Laboratory Medicine at Zhujiang Hospital, Southern Medical University. His research interest includes microbiome, clinical medicine and bioinformatics.

**Zhencheng Fang** is an assistant researcher in the Department of Laboratory Medicine at Zhujiang Hospital, Southern Medical University. His research interest includes genomics, bioinformatics and artificial intelligence.
