# Supplementary material for: metaProbiotics: a tool for mining probiotic from metagenomic binning data based on a language model
Source: Brief Bioinform. 2024 Mar 14;25(2):bbae085. doi: 10.1093/bib/bbae085 (PMC10940841; doi:10.1093/bib/bbae085)
Supplement: Supplementary_information_1_bbae085 [file supplementary_information_1_bbae085.docx]

**Supplementary information 1**

**
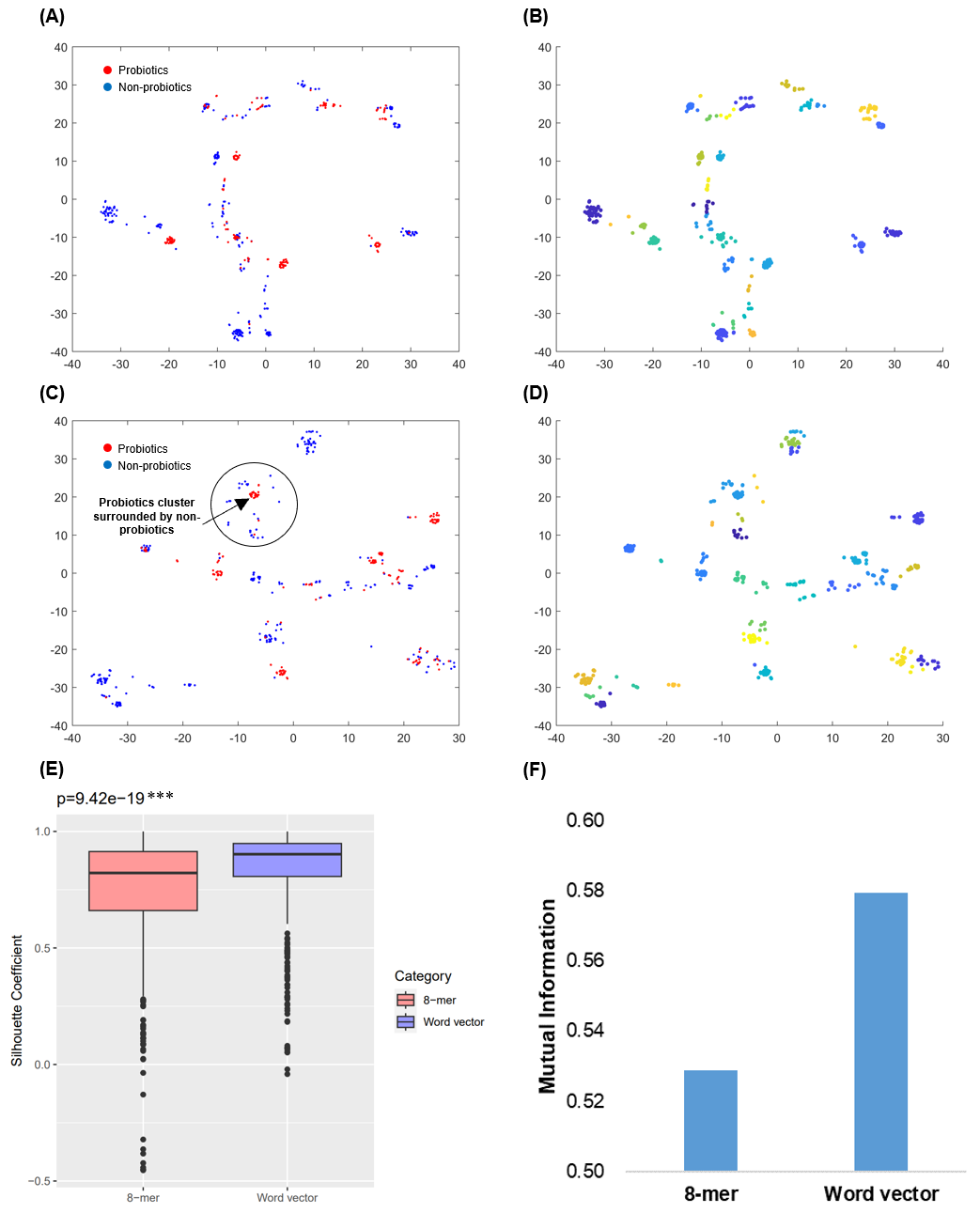
**

**Figure S1.** (A). t-SNE visualization of word vectors for probiotic and non-probiotic genomes. (B). Perform k-means clustering (k=30) on the dimensionality-reduced word vectors. (C). t-SNE visualization of 8-mer vector for probiotic and non-probiotic genomes. (D). Perform k-means clustering on the dimensionality-reduced 8-mer vectors. (E). Distribution of silhouette coefficients for each point in different models after clustering. (F). After clustering in different models, the mutual information between the assigned cluster categories of the genomes and their classification as probiotics.


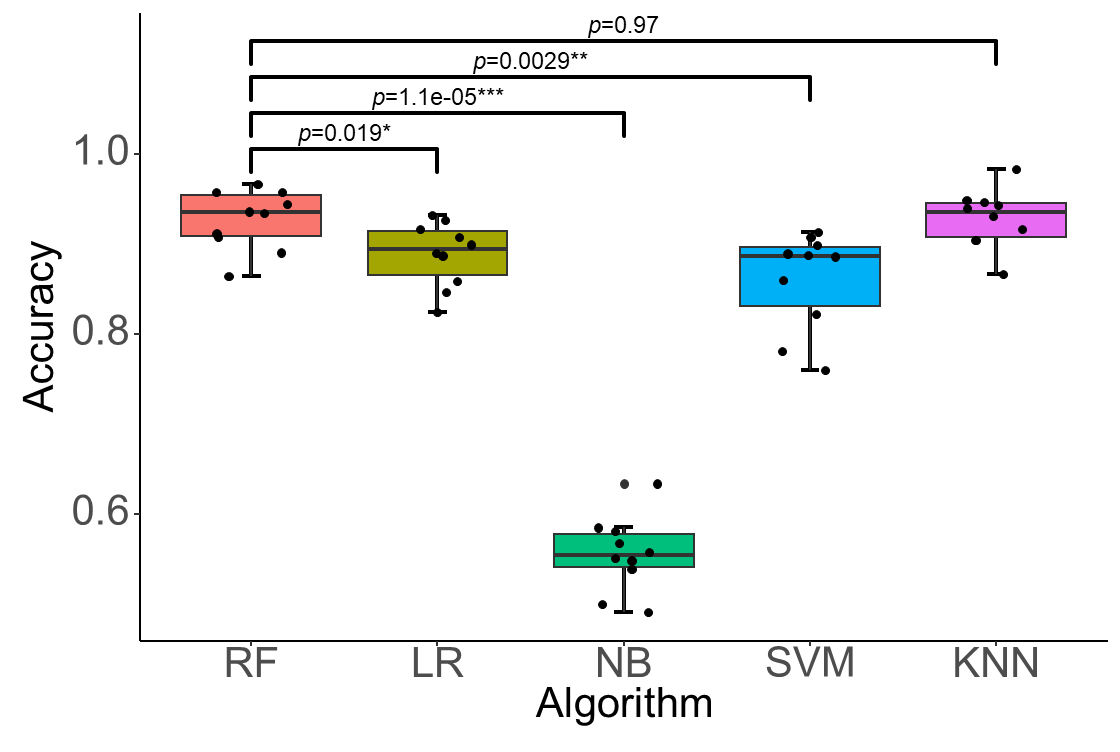


**Figure S2.** Evaluation of accuracy for various machine learning algorithms during 10-fold cross-validation on the training set. RF: random forest; LR: logistic regression; NB: naive Bayes; SVM: support vector machine; KNN: k-nearest neighbour.


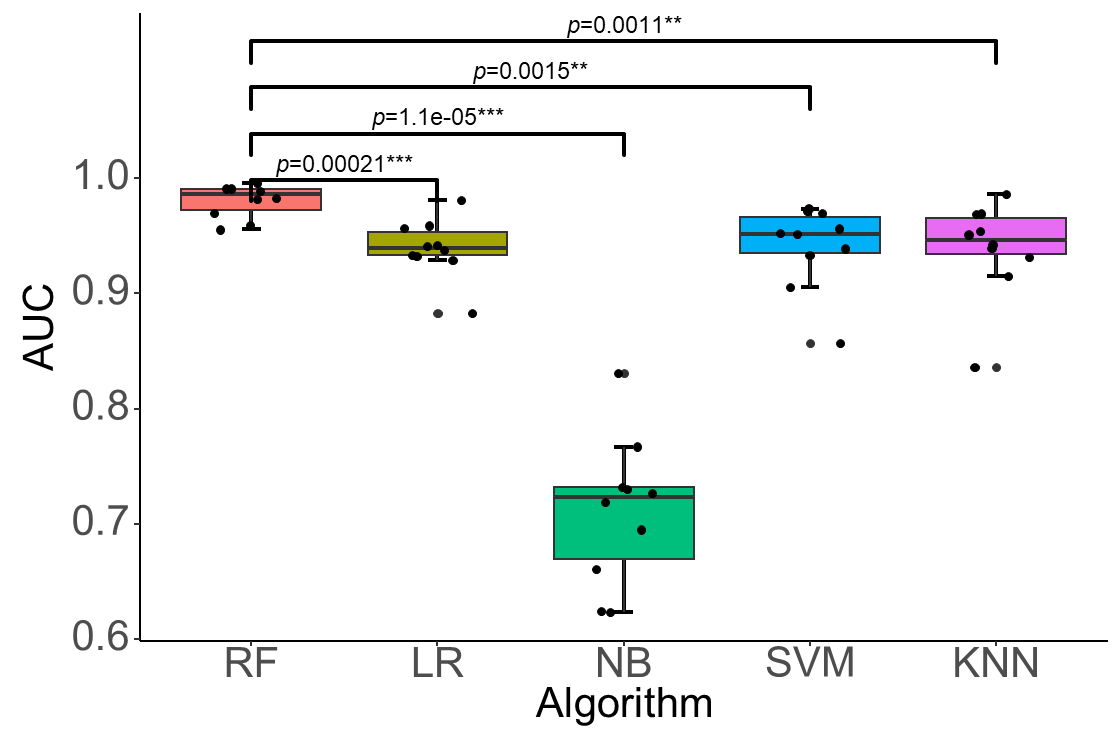


**Figure S3.** Evaluation of area under the curve (AUC) for various machine learning algorithms during 10-fold cross-validation on the training set. RF: random forest; LR: logistic regression; NB: naive Bayes; SVM: support vector machine; KNN: k-nearest neighbour.


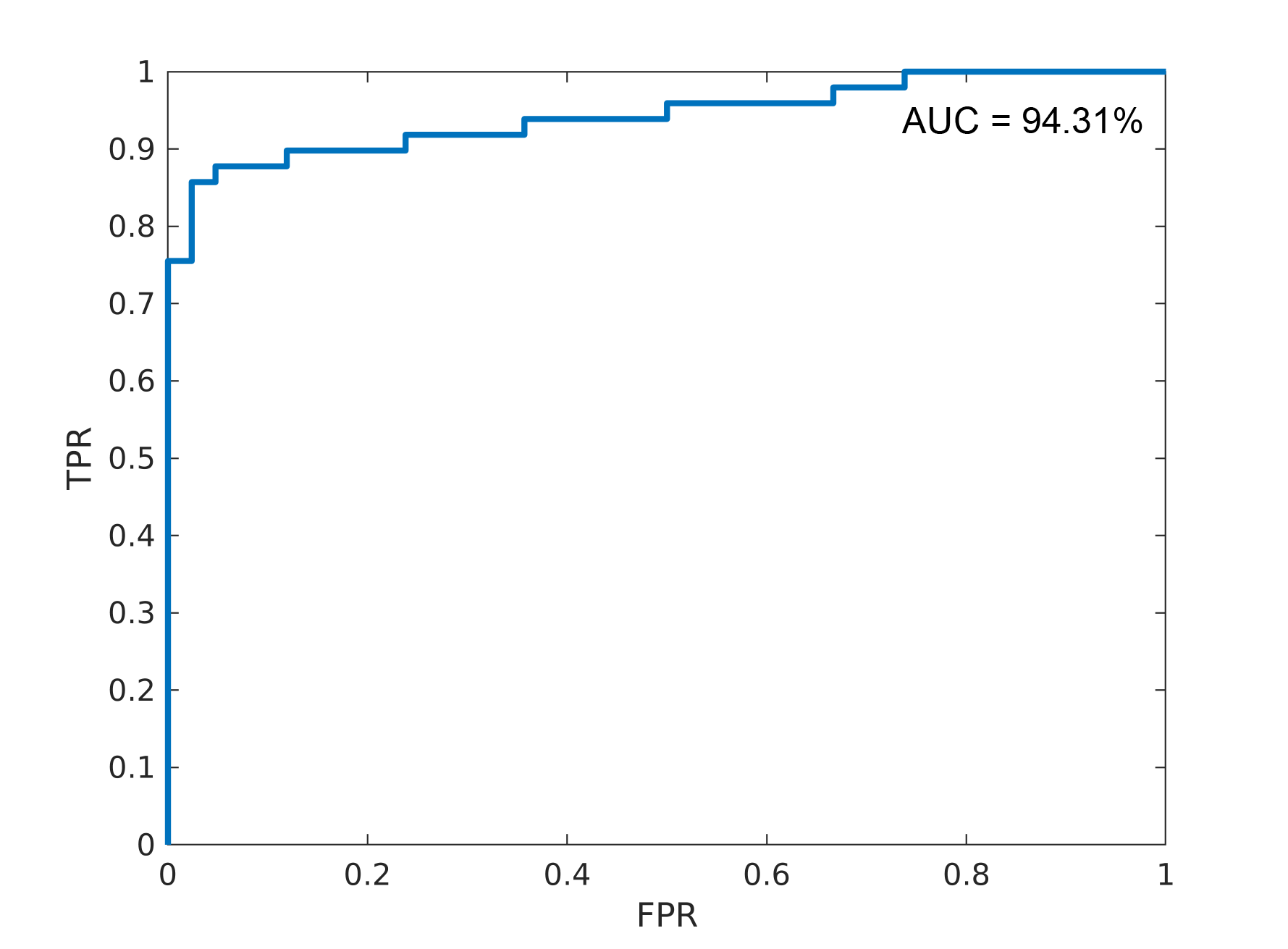


**Figure S4.** ROC curve for distinguishing probiotic and non-probiotic *Lactobacillus* bins in the benchmark test set using metaProbiotics.

**
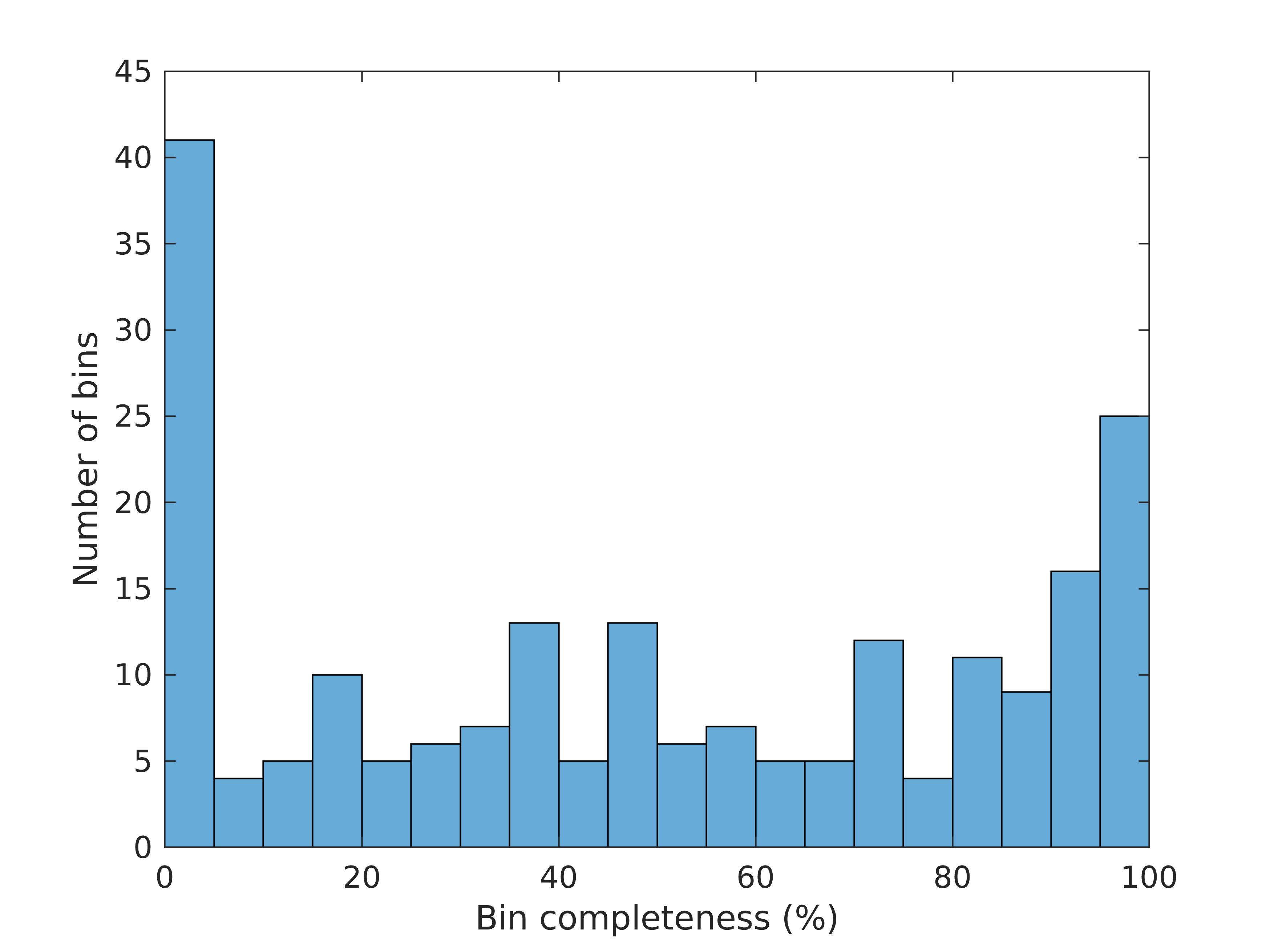
**

**Figure S5.** Distribution of bin counts with varying completeness levels in the LcZ group.


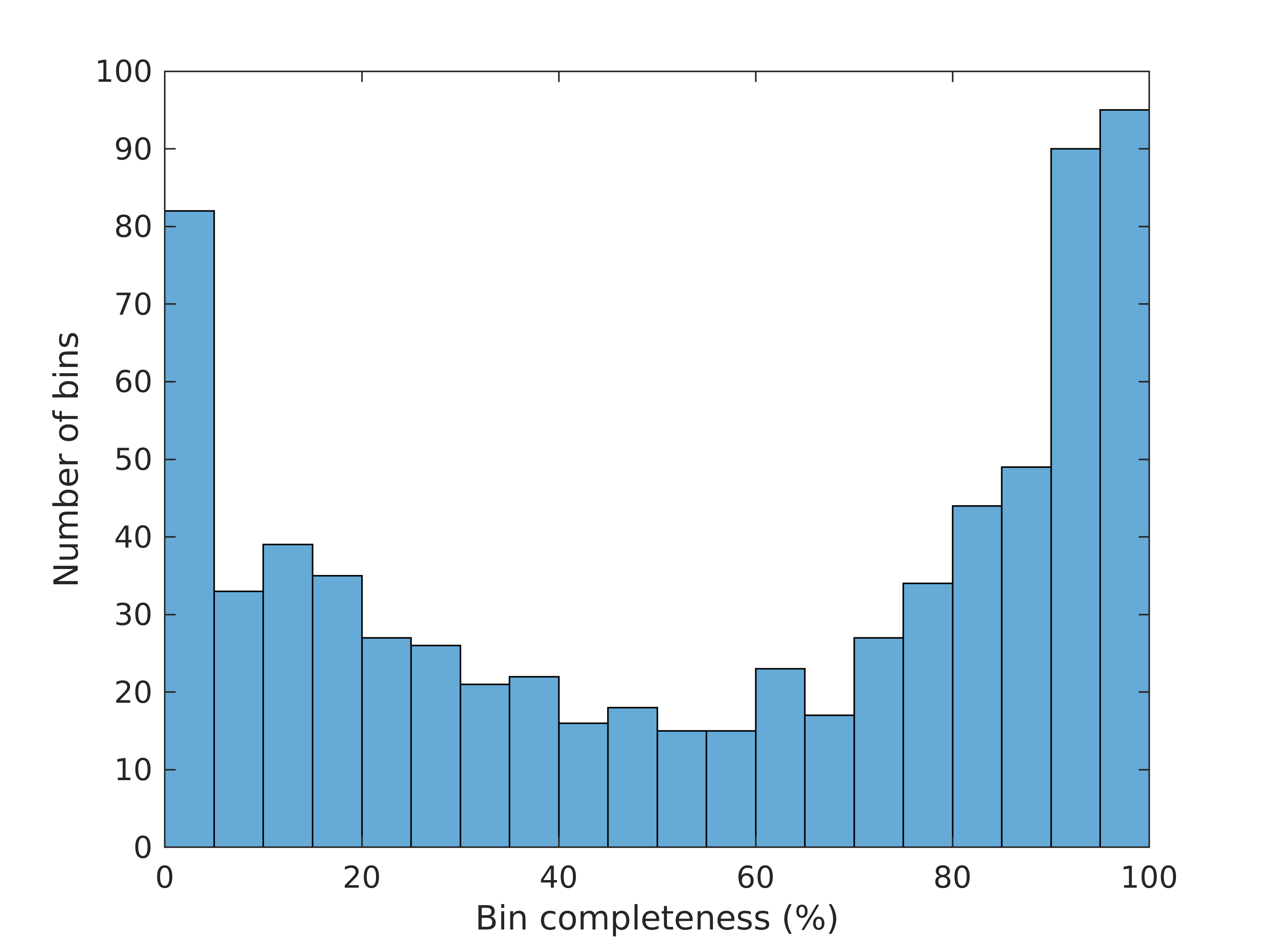


**Figure S6.** Distribution of bin counts with varying completeness levels in the BlA group.


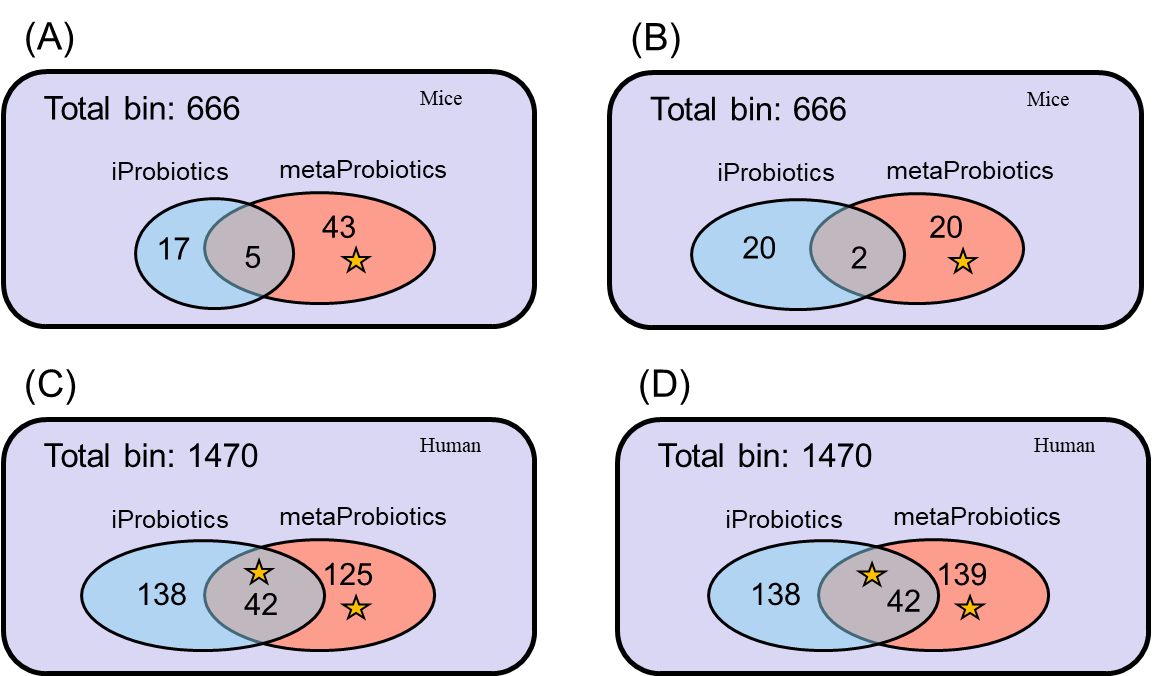


**Figure S7. Probiotic mining from mice and human metagenome derived from probiotic (*Lactiplantibacillus plantarum* HNU082) intervention cohorts.** (A). Similar to Fig. 3B in the main text. Venn diagram of the probiotic bins identified by the metaProbiotics and iProbiotics tools in the mice group. The yellow star indicates a bin with the highest ANI (97.44%) against the intervention strains. (B). Similar to Fig. 3C in the main text. Venn diagram for the mice group. We adjusted the decision threshold to allow metaProbiotics to identify the same number of probiotics as iProbiotics to perform a more quantitative comparison. (C). Similar to (A); Venn diagram for the mice group, yellow stars refer to bins with ANI higher than 98% against the intervention strains. (D). Similar to (B); Venn diagram for the human group after adjusting the decision threshold of metaProbiotics. In metaProbiotics’ predictions, two bins scored at the threshold, leading to one more bin being identified as a probiotic compared to iProbiotics.

Data description of Fig. S7: We utilized data from Huang *et al.*, in which they conducted interventions on human and mice populations using the probiotic strain *Lactiplantibacillus plantarum* HNU082. The samples’ accessions of human metagenome are: SRR10479015, SRR10479016, SRR10479017, SRR10479018, SRR10479020, SRR10479021, SRR10479022, SRR10479023, SRR10479024, SRR10479025, SRR10479026, SRR10479027, SRR10479028, SRR10479029, SRR10479031, SRR10479032, SRR10479033, SRR10479034, SRR10479035, SRR10479036, SRR10479037, SRR10479038, SRR10479039, SRR10479040, SRR10479042, SRR10479043, SRR10479044, SRR10479045, SRR10479046, SRR10479047, SRR10479048, SRR10479049, SRR10479050, SRR10479053, SRR10479054, SRR10479055, SRR10479056, SRR10479057, SRR10479059, SRR10479060, SRR10479061, SRR10479062; The samples’ accessions of mice metagenome are: SRR10479019, SRR10479030, SRR10479041, SRR10479051, SRR10479052, SRR10479058, SRR10479063, SRR10479064, SRR10479065, SRR10479066, SRR10479067, SRR10479068, SRR10479069, SRR10479070, SRR10479071, SRR10479072, SRR10479073, SRR10479074, SRR10479075, SRR10479076, SRR10479077, SRR10479078, SRR10479079, SRR10479080, SRR10479081, SRR10479082, SRR10479083, SRR10479084, SRR10479085, SRR10479086, SRR10479087, SRR10479088, SRR10479089, SRR10479090, SRR10479091, SRR10479092. The prediction results of iProbiotics and metaProbiotics are shown in Fig. S7. The metaProbiotics tool consistently identified bins from the intervention strain as probiotics, outperforming iProbiotics. The genomic data of the probiotic strain *Lactiplantibacillus plantarum* HNU082 was downloaded using the accession of SRR11971442, and was assembled by wtdbg (<https://github.com/ruanjue/wtdbg2>).

**
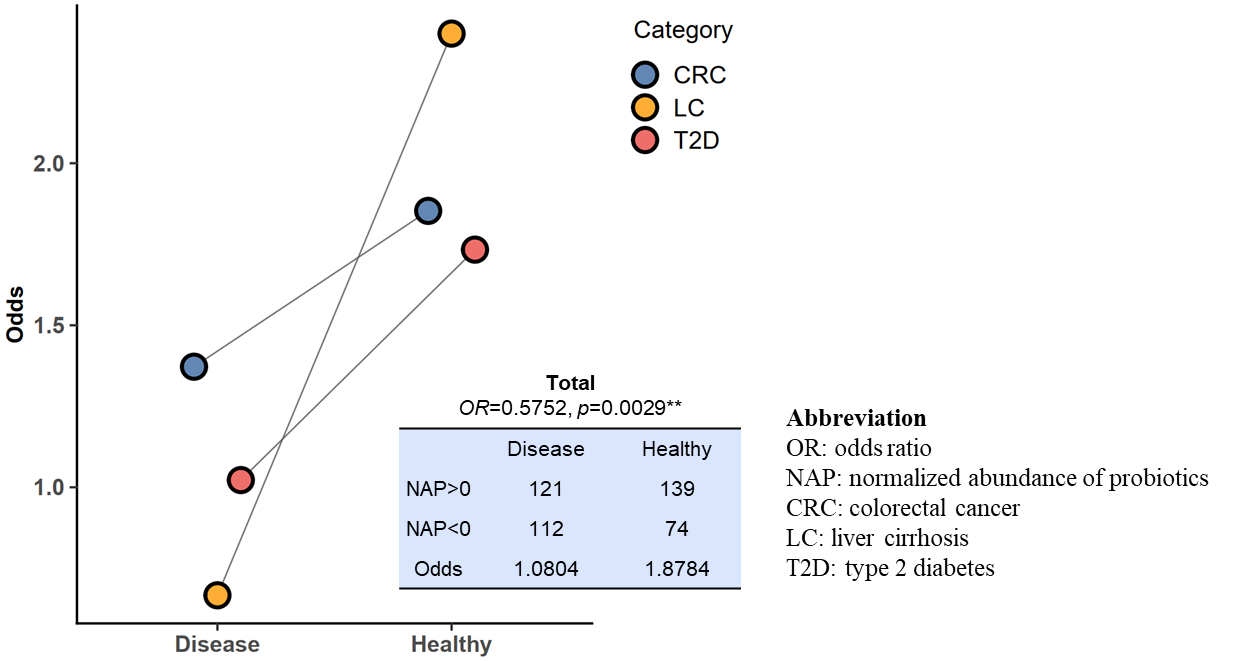
**

**Figure S8.** Using all predicted probiotics bin from LcZ and BlA group, we compared the overall normalized abundance of probiotics (NAP) between healthy individuals and those with specific diseases across different cohorts.


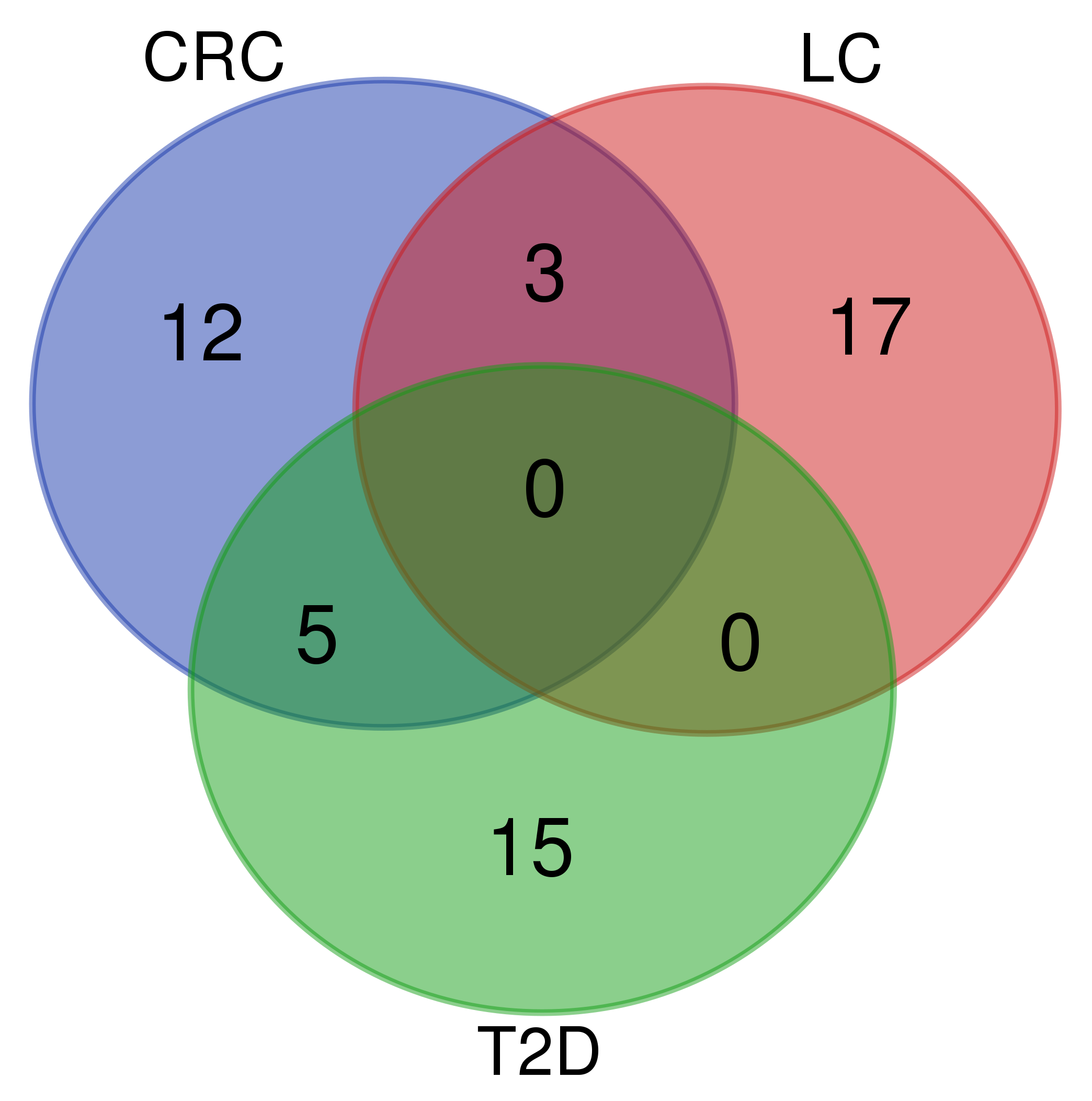


**Figure S9.** Utilizing the feature importance calculation based on out-of-bag (OOB) in random forests, we identified the top 20 bins of highest importance in health status prediction models constructed using different disease cohorts. We then observed the shared occurrence of these bins across various models through a Venn diagram.

**
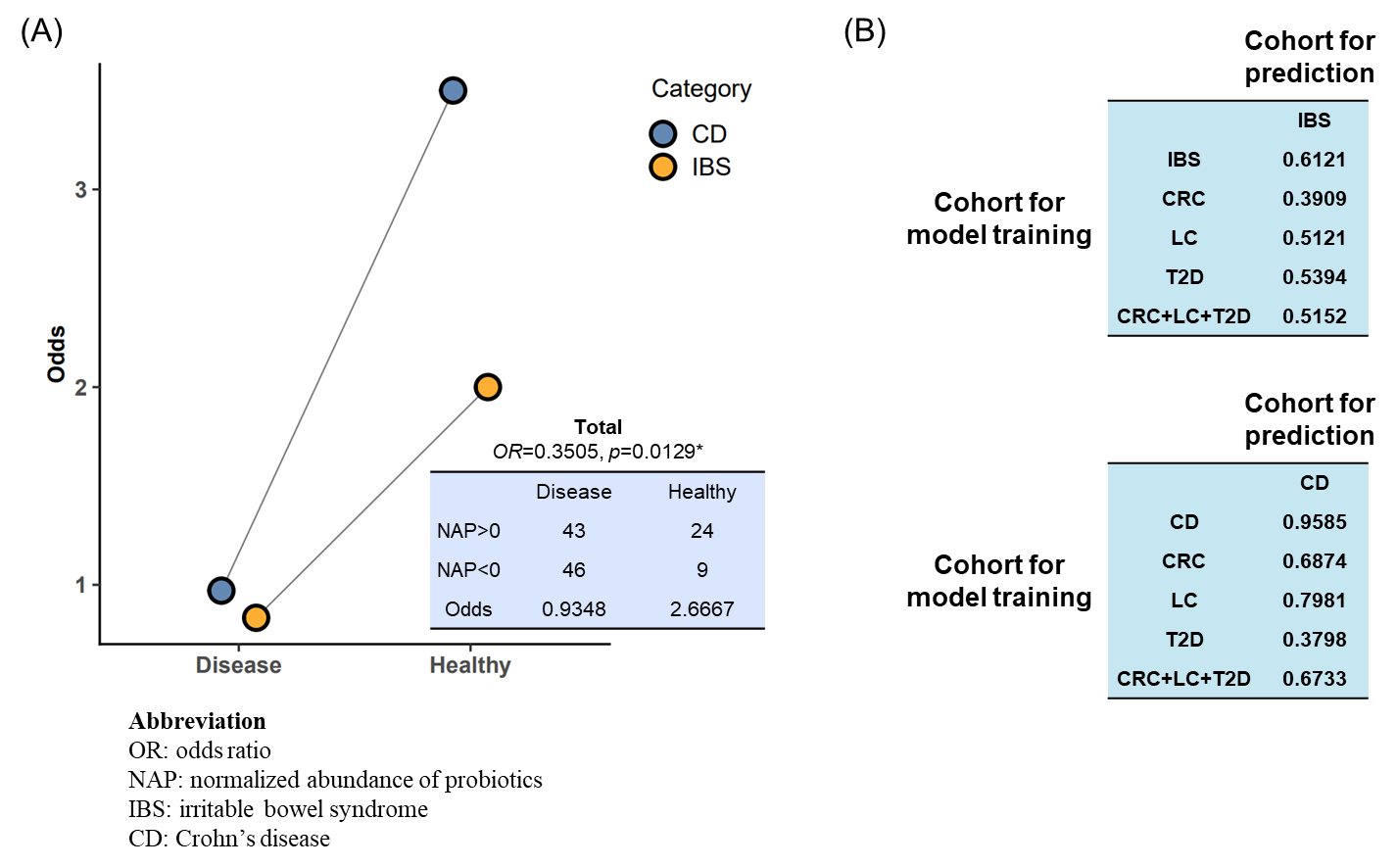
**

**Figure S10. Similar to Fig. 5 in the main text, we further analyzed the consistency and heterogeneity of probiotics in other distinct diseases, taking IBS and CD as examples.** (A) Similar to Fig. 5A in the main text, we compared the overall normalized abundance of probiotics (NAP) between healthy individuals and those with specific diseases across different cohorts. (B) Similar to Fig. 5C in the main text, we observed the AUC for probiotic abundance features-based health status prediction models within and across different diseases. When the cohort used for training was the same as the cohort used for prediction, we employed a leave-one-out approach to evaluate the AUC. We trained health status prediction models using the data from the three diseases cohorts mentioned in Fig. 5 of the main text, as well as the combined data of these three diseases. This training was followed by cross-disease health status predictions for IBS and CD.

**Table S2.** Employ metaProbiotics and iProbiotics for predicting draft genomes of *Lacticaseibacillus paracasei* sourced from NCBI.

| Accession | Max length (bp) | Min length (bp) | Average length (bp) | N50 (bp) | iProbiotics | metaProbiotics |
| --- | --- | --- | --- | --- | --- | --- |
| JAAXZG01.1 | 185763 | 1667 | 27044.00 | 64240 | Probiotic | Probiotic |
| CAUTBD01.1 | 326297 | 2874 | 36749.74 | 62313 | Probiotic | Probiotic |
| CAURZO01.1 | 349590 | 3869 | 83616.83 | 148434 | Probiotic | Probiotic |
| CAUVTX01.1 | 15068 | 2510 | 4478.15 | 4647 | Non-probiotic | Probiotic |
| CAUEKZ01.1 | 71883 | 2501 | 13323.94 | 18880 | Non-probiotic | Probiotic |
| CAUCPC01.1 | 54754 | 2521 | 8641.98 | 10382 | Non-probiotic | Probiotic |
| JAKECU01.1 | 61002 | 1517 | 9581.80 | 13728 | Non-probiotic | Probiotic |
| JAKECH01.1 | 51226 | 1540 | 9163.99 | 12604 | Non-probiotic | Probiotic |
| JAKECD01.1 | 74712 | 1508 | 8812.43 | 12331 | Non-probiotic | Probiotic |
| JAKEBF01.1 | 6196 | 1500 | 2392.79 | 2370 | Non-probiotic | Probiotic |

**Table S3.** Employ metaProbiotics and iProbiotics for predicting draft genomes of *Lactobacillus iners* sourced from NCBI.

| Accession | Max length (bp) | Min length (bp) | Average length (bp) | N50 (bp) | iProbiotics | metaProbiotics |
| --- | --- | --- | --- | --- | --- | --- |
| JAOBJG01.1 | 100032 | 1526 | 9617.41 | 19063 | Non-probiotic | Non-probiotic |
| JAOBIO01.1 | 64575 | 1534 | 8965.19 | 14555 | Non-probiotic | Non-probiotic |
| JAOBIK01.1 | 50321 | 1501 | 3945.64 | 4779 | Non-probiotic | Non-probiotic |
| JAOBIC01.1 | 37427 | 1506 | 5023.00 | 6603 | Non-probiotic | Non-probiotic |
| JAOBIB01.1 | 22885 | 1544 | 4260.33 | 5223 | Non-probiotic | Non-probiotic |
| JAOBHM01.1 | 20031 | 1513 | 2858.89 | 3016 | Non-probiotic | Non-probiotic |
| JAOBHQ01.1 | 12175 | 1512 | 3052.26 | 3403 | Non-probiotic | Non-probiotic |
| JAOBHS01.1 | 77535 | 1504 | 6647.73 | 9150 | Non-probiotic | Non-probiotic |
| JAOBHN01.1 | 13022 | 1550 | 3594.36 | 3961 | Non-probiotic | Non-probiotic |

**Table S4.** Employ metaProbiotics and iProbiotics for predicting draft genomes of *Bifidobacterium breve* sourced from NCBI.

| Accession | Max length (bp) | Min length (bp) | Average length (bp) | N50 (bp) | iProbiotics | metaProbiotics |
| --- | --- | --- | --- | --- | --- | --- |
| CALQQV02.1 | 47023 | 3011 | 7502.93 | 8527 | Non-probiotic | Non-probiotic |
| JAIHQQ01.1 | 16735 | 1006 | 2812.81 | 3365 | Non-probiotic | Probiotic |
| JAIHQY01.1 | 52700 | 1005 | 9016.86 | 16898 | Non-probiotic | Probiotic |
| JAIHRN01.1 | 113435 | 1018 | 13494.79 | 23389 | Non-probiotic | Probiotic |
| JAIHTW01.1 | 44476 | 1008 | 8283.63 | 14153 | Non-probiotic | Probiotic |
| JAIHUE01.1 | 28999 | 1001 | 3753.46 | 4817 | Non-probiotic | Probiotic |
| JAIJLE01.1 | 35865 | 1002 | 5061.84 | 8484 | Non-probiotic | Probiotic |
| JAIJMD01.1 | 27708 | 1000 | 3768.67 | 5504 | Non-probiotic | Probiotic |
| RKEL01.1 | 53974 | 2517 | 11605.87 | 16433 | Non-probiotic | Probiotic |

**Table S7.** The data quality of six bins that originate from the intervention probiotic strain from the BlA group, and their prediction status by metaProbiotics and iProbiotics.

| Bin ID | Max length (bp) | Min length (bp) | Average length (bp) | N50 (bp) | Completeness | Contamination | iProbiotics | metaProbiotics |
| --- | --- | --- | --- | --- | --- | --- | --- | --- |
| BlA_bin694 | 157432 | 1547 | 47495.23 | 91081 | 96.31% | 2.96% | Probiotics | Probiotics |
| BlA_bin727 | 301937 | 1501 | 10140.34 | 46288 | 100.00% | 281.82% | Probiotics | Probiotics |
| BlA_bin713 | 93677 | 1768 | 26019.16 | 42696 | 72.61% | 1.31% | Probiotics | Probiotics |
| BlA_bin536 | 42460 | 1516 | 7625.72 | 11685 | 25.86% | 0.00% | Non-probiotics | Probiotics |
| BlA_bin312 | 24682 | 1523 | 4988.85 | 6106 | 37.37% | 5.05% | Non-probiotics | Probiotics |
| BlA_bin531 | 38742 | 1527 | 4381.63 | 5263 | 30.57% | 5.59% | Non-probiotics | Probiotics |

Note: The completeness and contamination were estimated by the CheckM software.

**Table S8. Reported probiotic properties for probiotics predicted by metaProbiotics.** Only the genera that were not present in the probiotics training set are shown.

| Genus | Number of bins predicted in LcZ/BlA group^a^ | Reported probiotic property |
| --- | --- | --- |
| *Butyricicoccus* | 4/7 | (1) Improves NAFLD by altering the metabolites like SCFA and LPS^1^.  (2) Increases transepithelial resistance (TER) and attenuates the colitis^2^.  (3) Mitigates intestinal mucosal injury by increasing the SCFA^3^. |
| *Parabacteroides* | 4/0 | (1) Improves hepatic fibrosis by regulating hepatocyte pyroptosis and bile acid^4^.  (2) Ameliorates cardiovascular damage through strengthen the enhancing branched-chain amino acid catabolism^5^.  (3) Mitigates metabolic dysfunctions and obesity through the generation of succinate and secondary bile acids^6^. |
| *Phocaeicola* | 7/0 | (1) Protects against preeclampsia utilizing microbial processes such as the maltooligosaccharide transport mechanism and rhamnose transport system^7^.  (2) Resists LPS-induced acute intestinal injury and DSS-triggered colitis^8,9^.  (3) Promotes bile acid metabolism during the FMT process to prevent metabolic diseases^10^. |
| *Agathobaculum* | 4/5 | (1) Ameliorates cognitive impairment in Alzheimer's disease by regulating IGF-1 signalling^11^.  (2) Demonstrates neuroprotective capabilities in Parkinson's Disease by modulating the AKT/Nrf2/ARE signalling pathway^12^.  (3) Its butyrate derivatives may potentially prevent progressive multiple sclerosis^13^. |
| *Alistipes* | 0/9 | (1) Prolongs allograft survival by mechanisms such as inhibiting TNF production^14,15^.  (2) Promotes weight loss during physical activity by regulating metabolic pathways such as fatty acid β-oxidation and gluconeogenesis^16^.  (3) May prevent liver cirrhosis by enhancing the supplementation of energy sources^17^. |
| *Collinsella* | 0/16 | (1) Produces ursodeoxycholate to help alleviate infection and worsening symptoms of COVID-19^18^.  (2) Enhances the therapeutic effectiveness of PD-1 immunotherapy^19^.  (3) Degrades harmful byproducts of food processing by breaking down fructoselysine into lysine, formic acid, and acetic acid^20^. |
| *Ruminococcus* | 0/63 | (1) Enhances the therapeutic impact of CAR-T immunotherapy^21^.  (2) Reduces the risk of infantile atopic dermatitis by regulating butyrate levels^22^.  (3) Inhibits the infection of Vibrio cholerae primarily through the expression of luxS and Al-2 genes^23^. |

a. Genera with more than one bin in either group were shown.

**Reference of Table S3:**

1. Li Q, Liu W, Zhang H, Chen C, Liu R, Hou H, Luo Q, Yu Q, Ouyang H, Feng Y, Zhu W. α-D-1,3-glucan from Radix Puerariae thomsonii improves NAFLD by regulating the intestinal flora and metabolites. *Carbohydr Polym*. 2023 Jan 1;299:120197.
2. Eeckhaut V, Machiels K, Perrier C, Romero C, Maes S, Flahou B, Steppe M, Haesebrouck F, Sas B, Ducatelle R, Vermeire S, Van Immerseel F. Butyricicoccus pullicaecorum in inflammatory bowel disease. *Gut*. 2013 Dec;62(12):1745-52.
3. Shi H, Chang Y, Gao Y, Wang X, Chen X, Wang Y, Xue C, Tang Q. Dietary fucoidan of Acaudina molpadioides alters gut microbiota and mitigates intestinal mucosal injury induced by cyclophosphamide. *Food Funct*. 2017 Sep 20;8(9):3383-3393.
4. Zhao Q, Dai MY, Huang RY, Duan JY, Zhang T, Bao WM, Zhang JY, Gui SQ, Xia SM, Dai CT, Tang YM, Gonzalez FJ, Li F. Parabacteroides distasonis ameliorates hepatic fibrosis potentially via modulating intestinal bile acid metabolism and hepatocyte pyroptosis in male mice. *Nat Commun*. 2023 Apr 1;14(1):1829.
5. Qiao S, Liu C, Sun L, Wang T, Dai H, Wang K, Bao L, Li H, Wang W, Liu SJ, Liu H. Gut Parabacteroides merdae protects against cardiovascular damage by enhancing branched-chain amino acid catabolism. *Nat Metab*. 2022 Oct;4(10):1271-1286.
6. Wang K, Liao M, Zhou N, Bao L, Ma K, Zheng Z, Wang Y, Liu C, Wang W, Wang J, Liu SJ, Liu H. Parabacteroides distasonis Alleviates Obesity and Metabolic Dysfunctions via Production of Succinate and Secondary Bile Acids. *Cell Rep*. 2019 Jan 2;26(1):222-235.e5.
7. Lv LJ, Li SH, Wen JY, Wang GY, Li H, He TW, Lv QB, Xiao MC, Duan HL, Chen MC, Yi ZT, Yan QL, Yin AH. Deep metagenomic characterization of gut microbial community and function in preeclampsia. *Front Cell Infect Microbiol*. 2022 Sep 14;12:933523.
8. Li S, Wang C, Zhang C, Luo Y, Cheng Q, Yu L, Sun Z. Evaluation of the Effects of Different Bacteroides vulgatus Strains against DSS-Induced Colitis. *J Immunol Res*. 2021 May 29;2021:9117805.
9. Wang C, Xiao Y, Yu L, Tian F, Zhao J, Zhang H, Chen W, Zhai Q. Protective effects of different Bacteroides vulgatus strains against lipopolysaccharide-induced acute intestinal injury, and their underlying functional genes. *J Adv Res*. 2021 Jun 15;36:27-37.
10. Bustamante JM, Dawson T, Loeffler C, Marfori Z, Marchesi JR, Mullish BH, Thompson CC, Crandall KA, Rahnavard A, Allegretti JR, Cummings BP. Impact of Fecal Microbiota Transplantation on Gut Bacterial Bile Acid Metabolism in Humans. *Nutrients*. 2022 Dec 7;14(24):5200.
11. Go J, Chang DH, Ryu YK, Park HY, Lee IB, Noh JR, Hwang DY, Kim BC, Kim KS, Lee CH. Human gut microbiota Agathobaculum butyriciproducens improves cognitive impairment in LPS-induced and APP/PS1 mouse models of Alzheimer's disease. *Nutr Res*. 2021 Feb;86:96-108.
12. Lee DW, Ryu YK, Chang DH, Park HY, Go J, Maeng SY, Hwang DY, Kim BC, Lee CH, Kim KS. Agathobaculum butyriciproducens Shows Neuroprotective Effects in a 6-OHDA-Induced Mouse Model of Parkinson's Disease. *J Microbiol Biotechnol*. 2022 Sep 28;32(9):1168-1177.
13. Cox LM, Maghzi AH, Liu S, Tankou SK, Dhang FH, Willocq V, Song A, Wasén C, Tauhid S, Chu R, Anderson MC, De Jager PL, Polgar-Turcsanyi M, Healy BC, Glanz BI, Bakshi R, Chitnis T, Weiner HL. Gut Microbiome in Progressive Multiple Sclerosis. *Ann Neurol*. 2021 Jun;89(6):1195-1211.
14. Li Z, Rasic M, Kwan M, Sepulveda M, McIntosh C, Shastry V, Chen L, Finn P, Perkins D, Alegre ML. Oral administration of the commensal Alistipes onderdonkii prolongs allograft survival. *Am J Transplant*. 2023 Feb;23(2):272-277.
15. McIntosh CM, Chen L, Shaiber A, Eren AM, Alegre ML. Gut microbes contribute to variation in solid organ transplant outcomes in mice. *Microbiome*. 2018 May 25;6(1):96.
16. Wang K, Mehta RS, Ma W, Nguyen LH, Wang DD, Ghazi AR, Yan Y, Al-Shaar L, Wang Y, Hang D, Fu BC, Ogino S, Rimm EB, Hu FB, Carmody RN, Garrett WS, Sun Q, Chan AT, Huttenhower C, Song M. The gut microbiome modifies the associations of short- and long-term physical activity with body weight changes. *Microbiome*. 2023 May 30;11(1):121.
17. Shao L, Ling Z, Chen D, Liu Y, Yang F, Li L. Disorganized Gut Microbiome Contributed to Liver Cirrhosis Progression: A Meta-Omics-Based Study. *Front Microbiol*. 2018 Dec 18;9:3166.
18. Hirayama M, Nishiwaki H, Hamaguchi T, Ito M, Ueyama J, Maeda T, Kashihara K, Tsuboi Y, Ohno K. Intestinal Collinsella may mitigate infection and exacerbation of COVID-19 by producing ursodeoxycholate. *PLoS One*. 2021 Nov 23;16(11):e0260451.
19. Matson V, Fessler J, Bao R, Chongsuwat T, Zha Y, Alegre ML, Luke JJ, Gajewski TF. The commensal microbiome is associated with anti-PD-1 efficacy in metastatic melanoma patients. *Science*. 2018 Jan 5;359(6371):104-108.
20. Wolf AR, Wesener DA, Cheng J, Houston-Ludlam AN, Beller ZW, Hibberd MC, Giannone RJ, Peters SL, Hettich RL, Leyn SA, Rodionov DA, Osterman AL, Gordon JI. Bioremediation of a Common Product of Food Processing by a Human Gut Bacterium. *Cell Host Microbe*. 2019 Oct 9;26(4):463-477.e8.
21. Stein-Thoeringer CK, Saini NY, Zamir E, Blumenberg V, Schubert ML, Mor U, Fante MA, Schmidt S, Hayase E, Hayase T, Rohrbach R, Chang CC, McDaniel L, Flores I, Gaiser R, Edinger M, Wolff D, Heidenreich M, Strati P, Nair R, Chihara D, Fayad LE, Ahmed S, Iyer SP, Steiner RE, Jain P, Nastoupil LJ, Westin J, Arora R, Wang ML, Turner J, Menges M, Hidalgo-Vargas M, Reid K, Dreger P, Schmitt A, Müller-Tidow C, Locke FL, Davila ML, Champlin RE, Flowers CR, Shpall EJ, Poeck H, Neelapu SS, Schmitt M, Subklewe M, Jain MD, Jenq RR, Elinav E. A non-antibiotic-disrupted gut microbiome is associated with clinical responses to CD19-CAR-T cell cancer immunotherapy. *Nat Med*. 2023 Apr;29(4):906-916.
22. Sasaki M, Schwab C, Ramirez Garcia A, Li Q, Ferstl R, Bersuch E, Akdis CA, Lauener R; CK-CARE study group; Frei R, Roduit C. The abundance of Ruminococcus bromii is associated with faecal butyrate levels and atopic dermatitis in infancy. *Allergy*. 2022 Aug 2;77(12):3629–40.
23. Hsiao A, Ahmed AM, Subramanian S, Griffin NW, Drewry LL, Petri WA Jr, Haque R, Ahmed T, Gordon JI. Members of the human gut microbiota involved in recovery from Vibrio cholerae infection. *Nature*. 2014 Nov 20;515(7527):423-6.
