# Supplementary material for: metaProbiotics: a tool for mining probiotic from metagenomic binning data based on a language model
Source: Brief Bioinform. 2024 Mar 14;25(2):bbae085. doi: 10.1093/bib/bbae085 (PMC10940841; doi:10.1093/bib/bbae085)
Supplement: cover_letter_bbae085 [file cover_letter_bbae085.docx]

**Cover Letter**

Dear Editor,

Thank you very much for your previous E-mail on February 13, 2024 regarding our manuscript ‘‘metaProbiotics: a tool for mining probiotic from metagenomic binning data based on a language model’’ (Manuscript ID: BIB-23-2423.R1). We are very grateful for your acceptance decision. Following your instructions, we revised the key points, figure legend, figure format and a small typo.

We then report our revisions one by one as follows:

1. In Line 772-787, we have adjusted the Key points Section in bullet form.
2. In Line 1029-1109, we have provided a separate figure legend after the reference section of the main file.
3. In the legend of Table S8 in Supplementary Information 1 file, we have revised the sentence “Reported probiotic properties for probiotics predicted by metaProbiotic” to “Reported probiotic properties for probiotics predicted by metaProbiotics”.

In hoping that the above revision has met your requirements, we hereby resubmit our manuscript to *Briefings in Bioinformatics*. We thank you for your kind consideration.

Sincerely,

Zhencheng Fang, Ph.D.

Microbiome Medicine Center, Department of Laboratory Medicine, Zhujiang Hospital, Southern Medical University, Guangzhou, 510280, China

Email: [fangzc@smu.edu.cn](mailto:fangzc@smu.edu.cn)
